# cGAS–STING drives ageing-related inflammation and neurodegeneration

**DOI:** 10.1038/s41586-023-06373-1

**Published:** 2023-08-02

**Authors:** Muhammet F. Gulen, Natasha Samson, Alexander Keller, Marius Schwabenland, Chong Liu, Selene Glück, Vivek V. Thacker, Lucie Favre, Bastien Mangeat, Lona J. Kroese, Paul Krimpenfort, Marco Prinz, Andrea Ablasser

**Affiliations:** 1 Global Health Institute, Swiss Federal Institute of Technology Lausanne (EPFL), Lausanne, Switzerland; 2 Institute of Neuropathology, Faculty of Medicine, University of Freiburg, Freiburg, Germany; 3 Division of Endocrinology, Diabetology and Metabolism, Lausanne University Hospital, Lausanne, Switzerland; 4 Gene Expression Core Facility, Swiss Federal Institute of Technology Lausanne (EPFL), Lausanne, Switzerland; 5 Animal Modeling Facility, The Netherlands Cancer Institute, Amsterdam, The Netherlands; 6 Center for Basics in NeuroModulation (NeuroModulBasics), Faculty of Medicine, University of Freiburg, Freiburg, Germany; 7 Signalling Research Centres BIOSS and CIBSS, University of Freiburg, Freiburg, Germany; 8 Institute for Cancer Research (ISREC), Swiss Federal Institute of Technology Lausanne (EPFL), Lausanne, Switzerland

**Keywords:** Inflammation, Translational research, Innate immunity

## Abstract

Low-grade inflammation is a hallmark of old age and a central driver of ageing-associated impairment and disease^1^. Multiple factors can contribute to ageing-associated inflammation^2^; however, the molecular pathways that transduce aberrant inflammatory signalling and their impact in natural ageing remain unclear. Here we show that the cGAS–STING signalling pathway, which mediates immune sensing of DNA^3^, is a critical driver of chronic inflammation and functional decline during ageing. Blockade of STING suppresses the inflammatory phenotypes of senescent human cells and tissues, attenuates ageing-related inflammation in multiple peripheral organs and the brain in mice, and leads to an improvement in tissue function. Focusing on the ageing brain, we reveal that activation of STING triggers reactive microglial transcriptional states, neurodegeneration and cognitive decline. Cytosolic DNA released from perturbed mitochondria elicits cGAS activity in old microglia, defining a mechanism by which cGAS–STING signalling is engaged in the ageing brain. Single-nucleus RNA-sequencing analysis of microglia and hippocampi of a cGAS gain-of-function mouse model demonstrates that engagement of cGAS in microglia is sufficient to direct ageing-associated transcriptional microglial states leading to bystander cell inflammation, neurotoxicity and impaired memory capacity. Our findings establish the cGAS–STING pathway as a driver of ageing-related inflammation in peripheral organs and the brain, and reveal blockade of cGAS–STING signalling as a potential strategy to halt neurodegenerative processes during old age.

## Abstract (web summary)

The cGAS–STING signalling pathway is a critical driver of chronic inflammation and functional decline during ageing, and could be targeted to halt neurodegenerative processes during old age.

## Main

Ageing is characterized by decreased organismal fitness and it increases susceptibility to various diseases. Although compromised functionality of multiple homeostatic mechanisms can independently contribute to the ageing process, many of them converge on producing an aberrant inflammatory state that drives age-related decline^1^. Indeed, attenuation of age-related inflammation has emerged as a common mechanism by which (pharmacological) interventions into ageing exert their beneficial effects^4^.

Inflammation is typically triggered by the engagement of pattern-recognition receptors of the innate immune system. We and others have previously described a role of the cGAS–STING pathway in the regulation of cellular senescence—a hallmark of ageing^5–8^. However, whether the cGAS–STING pathway directly contributes to cellular senescence in human tissues or age-related inflammation and dysfunction in vivo remains unclear.

## STING elicits age-related inflammation

**Fig. 1:**
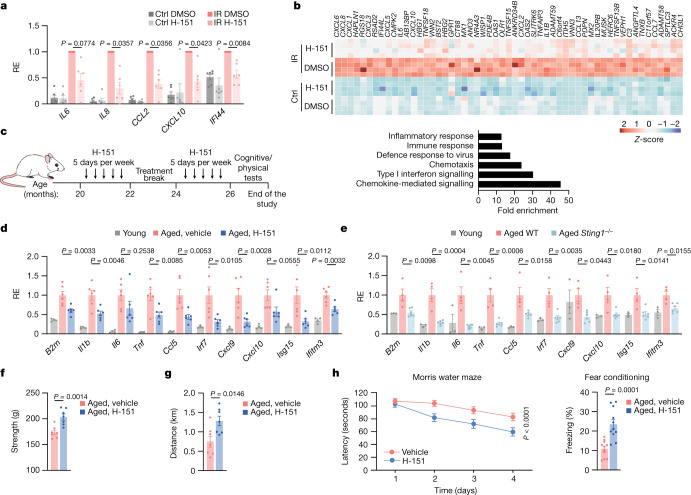
STING promotes low-grade inflammation and functional decline in aged mice. **a**,**b**, mRNA expression levels of proinflammatory genes and ISGs (**a**) and RNA-seq analysis (**b**) of human WI-38 fibroblasts irradiated (12 Gy, IR) or maintained at 5% O_2_ (Ctrl), and treated with H-151 (daily, 0.5 μM) or DMSO for 10 days when senescent (day 10 to 20). The relative expression (RE) was measured for each experiment (*n* = 6) relative to the induction level in the irradiated DMSO condition (**a**). **b**, The top 50 genes most upregulated after irradiation and suppressed after H-151 treatment (*n* = 4 experiments) (top), and a gene set enrichment analysis showing the fold enrichment based on the above list of genes (bottom). **c**, Schematic of the treatment of wild-type (WT) aged mice with H-151 related to data shown in **d** and **f**–**h**. **d**,**e**, Kidney mRNA expression levels of proinflammatory genes and ISGs in young (*n* = 4) and aged mice treated with or without H-151 (*n* = 6) (**d**) and of young (*n* = 3), aged WT (*n* = 4) and *Sting1*^*−/−*^ mice (*n* = 6) (**e**). Expression was measured relative to the average of aged vehicle-treated (**d**) or aged WT (**e**) mice. **f**,**g**, The physical condition of aged mice treated with or without H-151 (*n* = 7), evaluated by grip strength (**f**) and treadmill running distance (**g**). **h**, Cognitive function tests (*n* = 11 mice) were evaluated using the Morris water maze test (left, latency to reach the platform over multiple days) and fear conditioning (right, percentage of time spent freezing. *P* = 3 × 10^−5^. Data are mean ± s.e.m. *P* values were obtained using two-sided paired ratio Student’s *t*-tests (**a**), two-sided unpaired Student’s *t*-tests (**f**–**h** (right)), one-way analysis of variance (ANOVA) followed by Tukey’s multiple-comparison test (**d** and **e**) and ordinary two-way ANOVA (**h**, left). Source Data

**Extended Data Fig. 1:**
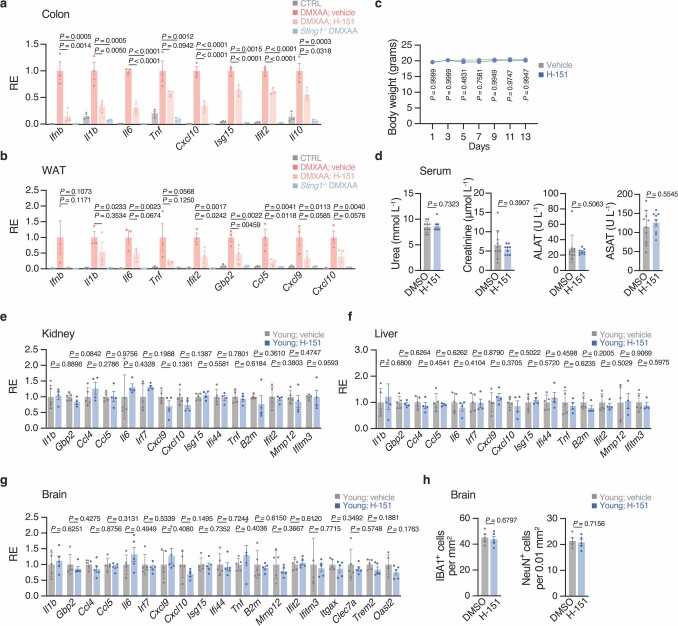
In vivo efficacy and tolerability measurements of H-151. **a**, **b** mRNA expression levels of proinflammatory and interferon-related genes in the colon (**a**) and the white adipose tissue (WAT, **b**) of WT and *Sting1*^*−/−*^ mice DMXAA-treated or not (CTRL), combined with vehicle or H-151 (*n* = 3), measured relative to the average induction levels of DMXAA vehicle-treated mice. Colon: H-151 *P* = 3 × 10^−6^ (Il6), 8 × 10^−5^ (Cxcl10), 9 × 10^−7^ (Isg15), 8 × 10^−5^ (Ifit2); *Sting1*^*−/−*^ *P* = 2 × 10^−7^ (Il6), 4 × 10^−6^ (Cxcl10), 5 × 10^−8^ (Ifit2). **c**–**h**, Young mice were treated daily with H-151 for 14 days. **c**, Body weight change with time from mice vehicle- or H-151-treated (*n* = 10). **d**, Serum creatinine, urea, ALAT, and ASAT levels in mice treated (*n* = 9) or not (*n* = 10) with H-151. **e**–**g**, mRNA expression levels of proinflammatory and interferon-related genes in the kidney (**e**), liver (**f**), and brain (**g**) of young mice treated (kidney, liver *n* = 4, brain *n* = 5) or not (*n* = 5) with H-151. Relative expression measured to the average induction levels of DMXAA vehicle-treated mice. **h**, Brain hippocampal counts of IBA1^+^ microglia (left) and NeuN^+^ neurons (right) from young mice treated or not with H-151 (*n* = 5). Data are mean ± s.e.m. *P* values were calculated by one-sided ordinary ANOVA followed by Tukey’s multiple comparisons tests (**a**,**b**), with two-sided Student’s unpaired *t*-test (**d**–**h**) or RM two-way ANOVA with Geisser-Greenhouse correction, followed by Sidak’s multiple comparisons tests (**c**). Source Data

**Extended Data Fig. 2:**
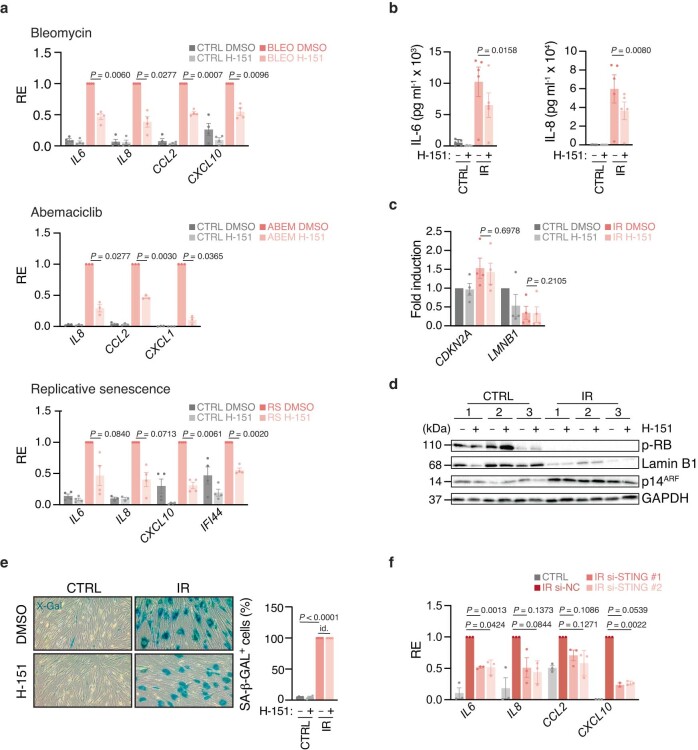
STING inhibition compromises the inflammatory phenotype of senescent cells. **a**, mRNA expression levels of proinflammatory and interferon-stimulated genes of human WI-38 fibroblasts rendered senescent by treatment with DNA metabolism inhibitor Bleomycin (*n* = 4 experiments), CDK4/6 inhibitor Abemaciclib (*n* = 3 experiments), repetitive passaging (*n* = 4 experiments) or maintained in 5% O_2_ (CTRL) and then treated with DMSO or H-151 (daily 0.5 μM for 10 days), measured for each experiment relative to the induction levels in the stimulated DMSO condition. **b**, Cytokine production measured by ELISA in the cultured medium of control and irradiated WI-38 cells treated with DMSO or H-151 (daily 0.5 μM from day 10 to day 20, from *n* = 5 experiments). **c**, mRNA expression levels of senescence characteristic genes in cells treated as in **b** (*n* = 4 experiments), measured for each experiment relative to the control DMSO condition. **d**, Western blot characterization of irradiated cells treated as in (**b**) harvested on day 20 (*n* = 3 experiments). **e**, Representative images and quantification of senescence-associated ß-galactosidase (SA-ß-GAL) staining from control and irradiated WI-38 cells treated as in (**b**), measured relative to total DAPI^+^ cells (*n* = 4 FOV, represents *n* = 3 experiments), *P* = 4 × 10^−5^. Id., identical values. **f**, mRNA expression levels of proinflammatory and interferon-stimulated genes in control and irradiated (12 Gy, IR) human BJ fibroblasts treated with si-NC or si-STING (72h, 10 days after irradiation). Relative expression normalized for each experiment (*n* = 3) to the induction in the irradiated si-NC condition. Data are mean ± s.e.m. *P* values were obtained with two-sided paired ratio Student’s *t*-test (**a**–**c**, **e**,**f**). Source Data

**Extended Data Fig. 3:**
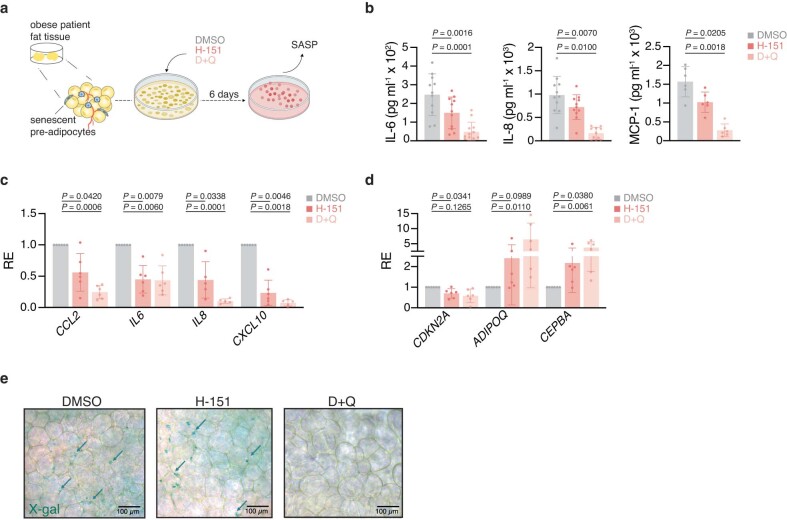
STING blockade decreases proinflammatory cytokine production in human adipose tissue. **a**, Schematic of omental adipose tissue explants studies. **b**, ELISA measurement, from individual patients, of IL-6 (*n* = 11), IL-8 (*n* = 11) and MCP-1 (*n* = 6) in the conditioned medium of tissue explants. **c**,**d**, mRNA expression levels of proinflammatory and interferon-stimulated genes (**c**) and adipose tissue functional genes (**d**), normalized per patient (*n* = 6) to the DMSO control condition. **e**, Representative images of senescence-associated β-galactosidase (SA-β-GAL) staining of omental fat tissue (*n* = 1 tissue, represents *n* = 11 patients), cleared with RapiClear. Arrows indicate examples of SA-β-GAL^+^ cells. Scale bars, 100 μm. Data are mean ± s.e.m. *P* values were obtained with two-sided paired ratio Student’s *t*-test (**b**–**d**). D + Q; senolytic drug combination Dasatinib and Quercetin. Source Data

To study the role of the cGAS–STING pathway in age-related phenotypes, we first tested whether acute inhibition of STING by the selective and well-tolerated small-molecular inhibitor H-151 (ref. ^9^) can suppress the inflammatory response of senescent cells, a paradigm in vitro model for studying age-related inflammation^10^ (Extended Data Fig. 1). Extending previous research^5–7^, H-151-mediated STING inhibition efficiently suppressed the induction of several proinflammatory genes and type I interferon (IFN)-stimulated genes (ISGs) in various contexts of senescence, without affecting other, non-inflammatory features of senescent cells^11^ (Fig. 1a,b and Extended Data Fig. 2a–e). RNA interference targeting of STING in fully senescent cells yielded similar results compared to pharmacological inhibition by H-151 (Extended Data Fig. 2f). Using explants of human adipose tissue from individuals with obesity, in whom the adipose tissue accumulates senescent preadipocytes^12^, we confirmed that STING inhibition by H-151 suppressed the release of proinflammatory signals from senescent cells at the tissue level (Extended Data Fig. 3). These data establish that inhibition of STING, both in cells and in human tissue, can block the inflammatory response of senescent cells—major contributors to inflammation during ageing^8^.

**Extended Data Fig. 4:**
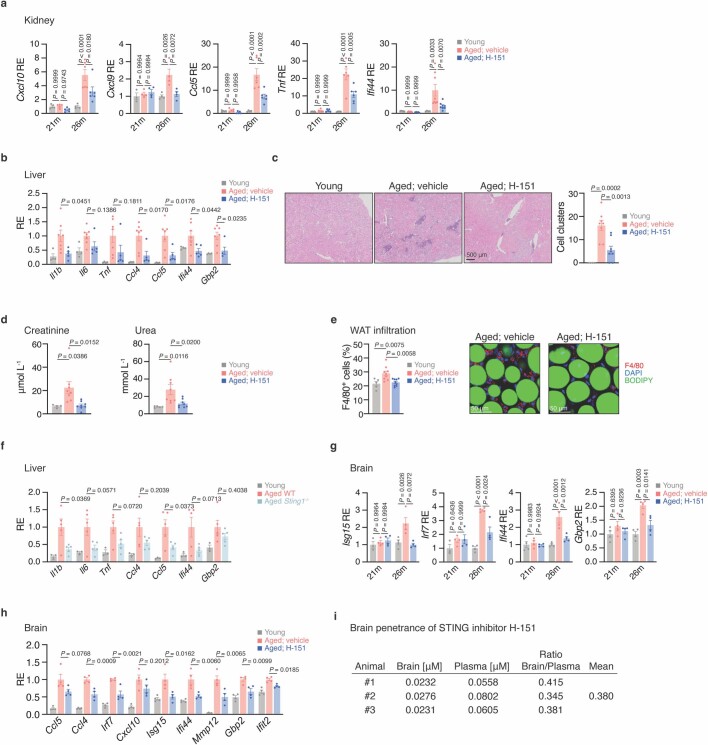
STING controls inflammatory signals in peripheral organs and the brain of aged mice. **a**, Kidney mRNA expression levels of proinflammatory and interferon-stimulated genes in young mice (21m *n* = 5 -*Cxcl9 n* = 3-, 26m *n* = 4), and mice at 21- and 26-months H-151-treated (21m *n* = 5 -*Cxcl9 n* = 4-, 26m *n* = 6 -*Cxcl9 n* = 4, *Ifi44 n* = 8-) or not (21m *n* = 5 -*Cxcl9 n* = 4-, 26m *n* = 6 -*Cxcl9 n* = 4, *Ifi44 n* = 8-). *P* = 3 × 10^-5^ (*Cxcl10*), 4 × 10^−7^ (*Ccl5*), 2 × 10^−7^ (*Tnf*). **b**, Liver mRNA expression levels of proinflammatory and interferon-stimulated genes in young (*n* = 4) and aged mice H-151-treated (*n* = 5) or not (*n* = 7). **c**, Representative histological images and quantification of inflammatory clusters in young (*n* = 4) and aged mice kidney H-151-treated or not (*n* = 8). Scale bar, 500 μm. **d**, Serum creatinine (left) and urea (right) levels in young (*n* = 4) and aged (*n* = 8) mice H-151-treated or not. **e**, Representative images and quantification of F4/80^+^ macrophages (red) in the white adipose tissue (WAT) of young (*n* = 5) and aged mice (*n* = 9) H-151-treated or not, relative to DAPI^+^ cells. Scale bars, 50 μm. **f**, Liver mRNA expression levels of proinflammatory and interferon-stimulated genes in young (*n* = 3) and aged WT/*Sting1*^*−/−*^ mice (*n* = 5), normalized to aged mice average. **g**, Brain mRNA expression levels of proinflammatory and interferon-stimulated genes in young (21m *n* = 4 -*Isg15*, *Irf7 n* = 3-, 26m *n* = 4), and mice at 21- and 26-months H-151-treated or not (*n* = 4). *P* = 4 × 10^−6^ (*Irf7*), 6 × 10^−5^ (*Ifi44*). **h**, Brain mRNA expression levels of proinflammatory and interferon-stimulated genes in young/aged mice H-151-treated or not (*n* = 4), normalized to aged vehicle-treated mice average. **i**, Brain penetrance measurements of H-151 in mice. Data are mean ± s.e.m. *P* values were calculated by one-way ANOVA followed by Tukey’s multiple comparisons tests (**a**–**h**). Source Data

We next used H-151 to block STING in aged mice (Fig. 1c). Consistent with previous research^13^, at a very old age (26 months), mice showed an inflammatory and type I IFN signature in the kidneys and the liver (Fig. 1d and Extended Data Fig. 4a,b). Notably, various ageing-related immune signature genes were significantly attenuated as a result of STING inhibition (Fig. 1d and Extended Data Fig. 4a,b). In parallel to the reduction in inflammatory cytokines, STING-inhibited animals showed reduced inflammatory cell accumulation in the kidneys, associated with reduced levels of kidney damage markers (creatinine, urea) and reduced numbers of macrophages in white adipose tissue compared with vehicle-treated aged mice (Extended Data Fig. 4c–e). Similarly, compared with old wild-type mice, aged *Sting1*^*−/−*^ mice displayed decreased levels of ageing-related immune markers, independently validating the effectiveness of H-151 to study the role of STING in mice in vivo (Fig. 1e and Extended Data Fig. 4f). We next examined whether attenuation of STING-dependent inflammation impacts the physical and cognitive function of old mice^14^. Both muscle strength and physical endurance, measured by grip strength and treadmill, respectively, improved in H-151-treated aged mice compared with the control mice (Fig. 1f,g). Testing hippocampal-dependent learning and memory performance, we observed that STING inhibition resulted in significantly improved spatial memory in the Morris water maze test and associative memory in the contextual-fear-conditioning test (Fig. 1h). Consistently, STING inhibition by H-151, a brain permeable compound, reduced the levels of immune-related signature genes in the brains of aged mice (Extended Data Fig. 4g–i). Together, these results establish STING as an important driver of ageing-associated inflammation, both in the periphery and the CNS, promoting frailty and cognitive decline.

## cGAS–STING affects brain ageing

**Fig. 2:**
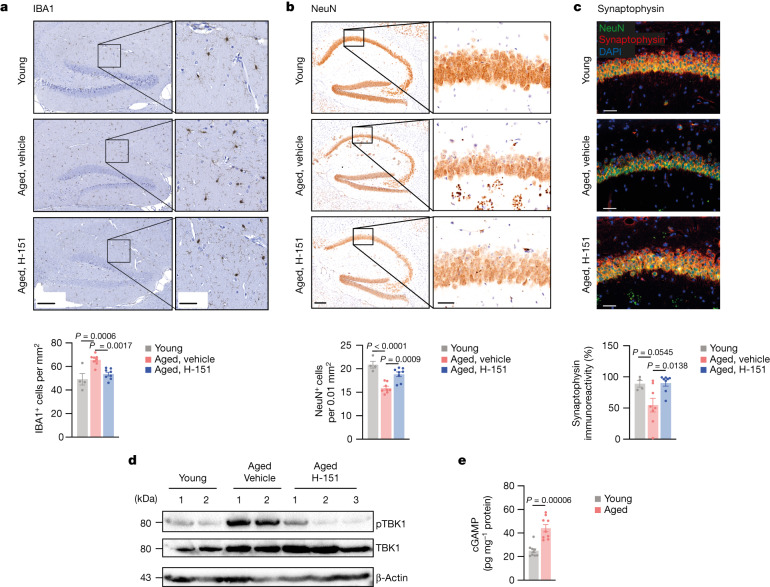
cGAS–STING activity drives degenerative processes in the aged brain. **a**–**c**, Representative images and quantification of hippocampal IBA1^+^ cells (**a**), NeuN^+^ cells (**b**) and synaptophysin intensity (**c**) in the CA1 region from brain sections of young (*n* = 4) and aged mice (*n* = 8) that were treated or not with H-151. Scale bars, 200 μm (**a** and **b** (left)), 50 μm (**a** and **b** (right) and **c**). *P* = 3 × 10^−5^. **d**, Western blot analysis of pTBK1 in the brain lysates of young mice (*n* = 2), aged mice (*n* = 2) and aged mice acutely treated with H-151 (daily for 5 consecutive days, *n* = 3). **e**, cGAMP production measured by enzyme-linked immunosorbent assay (ELISA) in brain lysates of young and aged mice (*n* = 9). Data are mean ± s.e.m. *P* values were calculated using ordinary one-way ANOVA followed by Tukey’s multiple-comparison tests (**a**–**c**) or two-sided unpaired Student’s *t*-tests (**e**). Source Data

**Extended Data Fig. 5:**
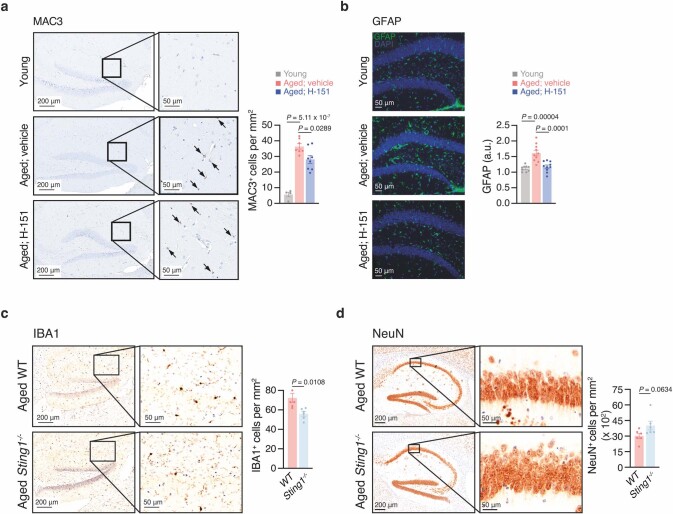
STING activation disrupts brain cell homeostasis in aged mice. **a**, Representative images and quantification of hippocampal MAC3 in brain sections of young (*n* = 4) and aged mice vehicle- (*n* = 7) or H-15-treated (*n* = 8). Arrows indicate MAC3^+^ cells. Scale bars, 200 μm (left), 50 μm (right). **b**, Representative images and quantification of hippocampal GFAP in brain sections of young (*n* = 9) and aged mice (*n* = 10) vehicle- or H-151-treated. Scale bar, 50 μm; a.u., arbitrary units. **c**,**d**, Representative images and quantification of hippocampal IBA (**c**) and NeuN (**d**) in the CA1 region of aged WT (IBA1, *n* = 5; NeuN, *n* = 6) and *Sting1*^*−/−*^ mice (*n* = 6). Scale bars, 200 μm (left), 50 μm (right). Data are mean ± s.e.m. *P* values were calculated by one-way ANOVA followed by Tukey’s multiple comparisons tests (**a**,**b**) or two-sided Student’s unpaired *t*-test (**c**,**d**). Source Data

The signalling mechanisms underlying the initiation of maladaptive inflammatory and type I IFN responses in the ageing brain are incompletely understood^15,16^. We therefore focused on understanding the contribution of the cGAS–STING pathway to brain ageing. Histopathological examination of the hippocampal parenchyma revealed microgliosis^17^ in aged mice, which was reduced after STING inhibition (Fig. 2a). Concomitantly, aged microglia showed less expression of the lysosomal marker MAC3 when STING was inhibited (Extended Data Fig. 5a). The immunoreactivity of astrocytes in the hippocampi of aged mice was also mitigated by H-151 (Extended Data Fig. 5b). Moreover, STING inhibition protected mice from the loss of neurons in the CA1 region of the hippocampus and increased local levels of synaptophysin, a marker for synaptic activity (Fig. 2b,c). We confirmed that, compared with aged wild-type mice, aged *Sting1*^*−/−*^ mice displayed reduced microglial accumulation along with increased neuron density in the hippocampal area (Extended Data Fig. 5c,d). Thus, these results demonstrate that STING affects brain homeostasis in aged mice.

During ageing, systemic factors can impair adult neurogenesis and contribute to cognitive dysfunction^18^. To define whether the observed effects result from brain-intrinsic STING activity, we analysed biomarkers of STING signalling in whole-brain lysates of aged mice. The levels of phosphorylated TBK1 (Ser172; pTBK1)—the major kinase responsible for signal transduction downstream of STING^19,20^—were increased in aged mouse brains (Fig. 2d). Acute STING inhibition in aged mice led to a reduction of the pTBK1 signal, indicating that the STING–TBK1 signalling axis is locally engaged in the aged brain (Fig. 2d). In DNA sensing, STING is activated by 2′3′-cGAMP (cGAMP) produced endogenously by cGAS^21–25^. We detected robust cGAMP production in brain lysates from old mice, but not young mice (Fig. 2e). Together, these data reveal that STING is activated inside the aged brain of mice and implicate aberrant cGAS activity upstream of STING signalling during ageing.

## STING activity in aged microglia

**Fig. 3:**
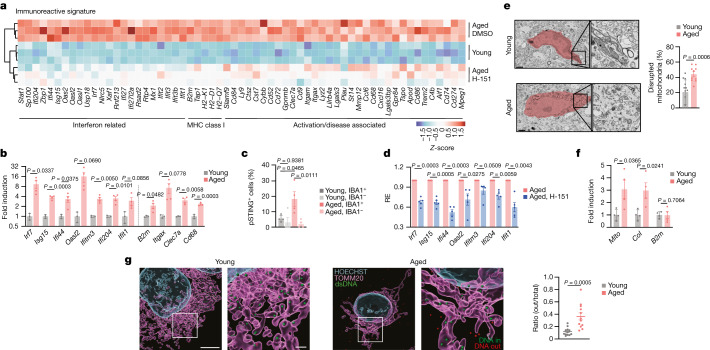
Aberrant cGAS–STING activation in microglia of aged mice involves mtDNA. **a**, Differential gene expression from bulk hippocampus RNA-seq analysis of young and aged mice treated with or without H-151 (*n* = 3). The heat map shows type I IFN, MHC class I and microglial activation/disease-associated genes from the total list of significant DEGs (Extended Data Fig. 6a; false-discovery rate (FDR) ≤ 0.01, |log_2_[fold change (FC)]| ≥ 0.6). **b**, mRNA expression levels of immunoreactive genes, ISGs and activation markers in primary microglia isolated from young (*n* = 3) and aged (*n* = 4) mice. **c**, Confocal imaging quantification of pSTING staining in young and aged hippocampal sections (average from 100–200 cells per mouse, *n* = 4), differentially quantified in IBA1^−^ and IBA1^+^ cells. **d**, mRNA expression levels of immunoreactive genes and ISGs in microglia from aged mice treated or not with H-151. *n* = 5. **e**, Transmission electron microscopy images representing age-related microglial morphological differences and the percentage of disrupted mitochondria per hippocampal microglia in young (*n* = 13) and aged (*n* = 10) cells, randomly selected from 3 mice per condition. Scale bars, 1 μm. **f**, Cytosolic expression levels of *Mito* (mitochondrial DNA sequence), *CoI* and *B2m* in microglia isolated from young and aged mice (*n* = 4). **g**, Representative 3D reconstructions from Airyscan images and quantification of cytosolic DNA foci outside the mitochondria in microglia isolated from young and aged mice. The ratio of DNA foci outside the mitochondria was measured for each cell relative to the total counts of cytosolic foci (inside (green) and outside (red)). *n* = 12 cells, from 3 mice. Scale bars, 5 μm (top) and 1 μm (bottom). Data are mean ± s.e.m. *P* values were calculated using two-sided unpaired Student’s *t*-tests (**b** and **d**–**g**) and one-way ANOVA followed by Tukey’s multiple-comparison test (**c**). Source Data

**Extended Data Fig. 6:**
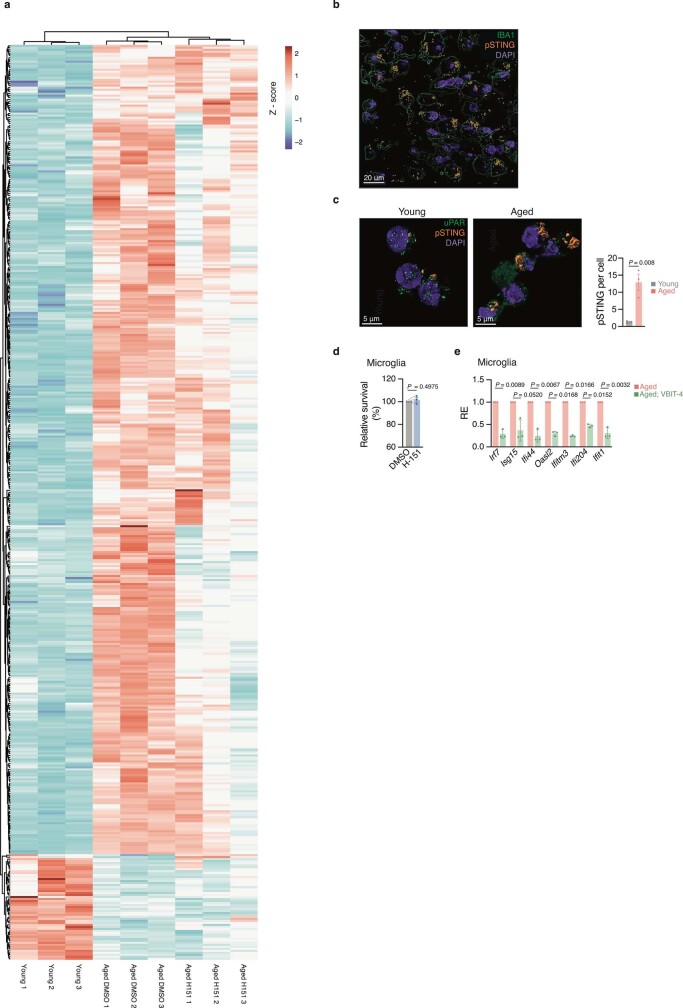
STING-dependent effects in aged microglia. **a**, Heatmap depicting aging responsive genes (459 genes, FDR ≤ 0.01, Log2FC ≥ 0.6 *n* = 3 per condition). Colours are representative of normalized counts per million. Genes and samples are ordered by unsupervised clustering. **b**, Representative confocal image of pSTING staining in aged hippocampi sections (*n* = 1, represents *n* = 4 mice). Scale bar, 20 μm. **c**, Representative confocal images and quantification of pSTING foci in microglia cultured from young and aged mice (average from 20 cells per mouse, *n* = 3). Scale bars, 5 μm. **d**, Relative survival of primary microglia treated with H-151 for 24 h, measured by Cell titre blue (CTB) assay (*n* = 3 mice). **e**, mRNA expression levels of immunoreactive and interferon-stimulated genes in primary microglia from aged mice treated or not VBIT-4 (10 μm) for 4 days (*n* = 3). Data are mean ± s.e.m. *P* values were calculated by two-sided Student’s unpaired (**c**,**d**) or two-sided Student’s paired (**e**) *t*-test. uPAR, urokinase-type plasminogen activator receptor. Source Data

To uncover potential mechanisms underlying cGAS–STING activation in the brain, we performed bulk RNA-sequencing (RNA-seq) analysis of hippocampi from young adult mice and old mice that were treated or not with H-151. Transcriptional analysis revealed 459 significantly differentially expressed genes (DEGs) between young and old hippocampi, with a substantial portion of these genes involved in innate immunity, including genes related to type I IFN signalling and microglial function (Fig. 3a and Extended Data Fig. 6a). Induction of several DEGs was attenuated in the group receiving H-151, which clustered together with young mice (Fig. 3a and Extended Data Fig. 6a). Befitting their central role in shaping the immune state of the CNS^26^, primary microglia isolated from the brain of old mice featured a similar increase in the expression of innate immune genes (Fig. 3b). We therefore considered that the neurodegenerative effects of STING in brain ageing involve microglia. To investigate the STING response within microglia, we visualized phosphorylated STING (Ser365; pSTING), a direct marker of STING activity^27^, in the hippocampal area. Relative to young mice, pSTING foci were enriched in aged hippocampi and, most of the pSTING signal derived from IBA1^+^ microglia (Fig. 3c and Extended Data Fig. 6b,c). Ex vivo experiments with aged microglia confirmed STING-dependent expression of type I IFN and proinflammatory genes (Fig. 3d). Collectively, these results demonstrate STING activity in microglia of old mice, which directs innate immune activation in the ageing brain.

## mtDNA activates cGAS in aged microglia

**Extended Data Fig. 7:**
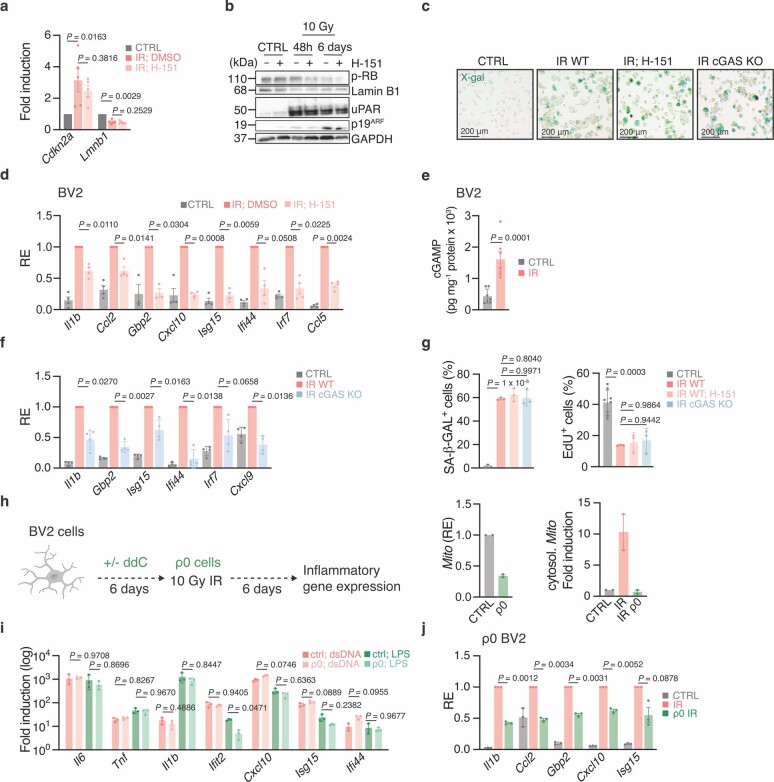
Senescent microglial BV2 cells display cGAS–STING-dependent innate immune activation. **a**–**d**, BV2 cells were irradiated (10 Gy, IR), then DMSO- or H151-treated (daily, 1 μM, day 4-6 post-irradiation). mRNA expression levels of senescence markers (*n* = 5 experiments) (**a**), Western blot characterization (**b**), and senescence-associated-β-galactosidase staining (*n* = 1, represents *n* = 3 experiments) (**c**). Scale bars, 200 μm. mRNA expression levels of proinflammatory and interferon-stimulated genes (**d**) in control and irradiated, DMSO- or H-151-treated cells, measured for each experiment (*n* = 4) relative to irradiated DMSO-treated cells. **e**, cGAMP levels in cell lysates of control and irradiated BV2 cells (*n* = 7 experiments). **f**, mRNA expression levels of proinflammatory and interferon-stimulated genes in control and irradiated WT and cGAS-KO BV2 cells (*n* = 4 experiments). **g**, Senescence-associated-β-galactosidase (left, *n* = 3 FOV) and % EdU^+^ cells (right) from control (*n* = 8 FOV) and irradiated (*n* = 3 FOV) WT BV2, H-151-treated or not as in (**a**) and cGAS-KO BV2 (*n* = 3 FOV), represents *n* = 3 experiments. **h**, Experimental set-up for the analysis of mtDNA-depleted BV2 cells (ρ0 BV2) (left). Mitochondrial DNA sequence *Mito* levels in ρ0 BV2 whole cell lysate (middle), and in the cytosol after irradiation (right) (*n* = 2 experiments). **i**, mRNA expression levels of proinflammatory and interferon-stimulated genes in ρ0 BV2 cells stimulated with LPS or dsDNA 90mer transfection (*n* = 3 experiments). **j**, mRNA expression levels of proinflammatory and interferon-stimulated genes in control and irradiated ρ0 BV2 cells, measured for each experiment (*n* = 3) relative to irradiated untreated (IR) cells. Data are mean ± s.e.m. *P* values were obtained with two-sided paired ratio Student’s *t*-test (**a**,**d**,**e**,**j**), two-sided unpaired Student’s *t*-test (**f**), and one-way ANOVA followed by Tukey’s multiple comparisons tests (**g**,**i**). uPAR, urokinase-type plasminogen activator receptor. Source Data

Mitochondrial DNA (mtDNA) is a central activator of cGAS–STING signalling^28^, and disrupted mitochondrial homeostasis is a hallmark of ageing and neurodegenerative disease^29^. On the basis of transmission electron microscopy, the mitochondria in aged microglia appeared misshapen and lacked their characteristic internal structure (Fig. 3e). To establish whether mtDNA is released into the cytosol of aged microglia, we analysed the cytosolic DNA content of primary microglia using quantitative PCR with reverse transcription (RT–qPCR). Compared with microglia from young mice, aged microglia displayed an increased abundance of mtDNA, but not genomic DNA species (Fig. 3f). Super-resolution Airyscan imaging of aged microglia affirmed prominent cytosolic accumulation of mtDNA nucleoids adjacent to the mitochondria outer membrane, which was largely absent in the cytosol of young microglia (Fig. 3h). We next sought to address whether aberrant mtDNA contributes to the inflammatory phenotype of aged microglia. Previous research has implicated voltage-dependent ion channel (VDAC) 1/3 oligomers in the cytosolic release of mtDNA (ref. ^30^). Ex vivo treatment of aged microglia with VBIT-4, an inhibitor of VDAC oligomerization, suppressed several type I IFN and proinflammatory genes (Extended Data Fig. 6e). An in vitro cellular senescence model corroborated a role of mtDNA in triggering cGAS-dependent inflammatory responses in microglia-derived mouse BV2 cells (Extended Data Fig. 7). Together, these data indicate a role of mtDNA in directing inflammatory responses in aged microglia and define a molecular model of cGAS–STING activation during ageing and senescence.

## Effect of microglial cGAS on the brain

**Fig. 4:**
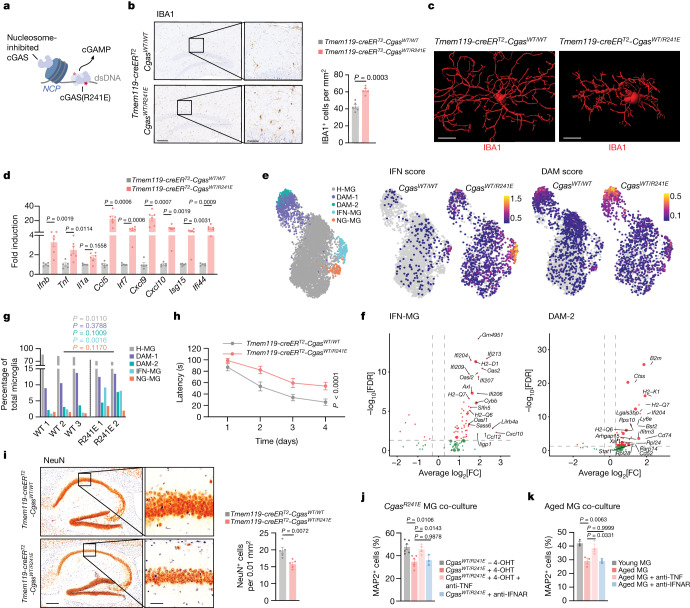
Selective engagement of cGAS promotes age-associated microglial states and features of neurodegeneration. **a**, Schematic of the nucleosome-binding-defective cGAS-mutant activation. **b**, Representative images and quantification of hippocampal IBA1 staining of *Tmem119-creER*^*T2*^*-Cgas*^*WT/WT*^ and *Tmem119-creER*^*T2*^*-Cgas*^*WT/R241E*^ mice. *n* = 5. Scale bars, 200 μm (left) and 50 μm (right). **c**, Representative IBA1^+^ microglia reconstructed by IMARIS. Scale bars, 10 μm. **d**, Brain mRNA expression levels of proinflammatory genes and ISGs from *Tmem119-creER*^*T2*^*-Cgas*^*WT/WT*^ (*n* = 5) and *Tmem119-creER*^*T2*^*-Cgas*^*WT/R241E*^ (*n* = 6) mice. **e**, Uniform manifold approximation and projection (UMAP) plots visualizing microglial single nuclei, coloured by cell identity (left, homeostatic microglia (H-MG); disease-associated microglia (DAM-1/2); IFN-associated microglia (IFN-MG); neurodegenerative-associated microglia (ND-MG)), and IFN/DAM gene expression scores split by *Cgas* genotype (right). Colour scale bars denote the gene burden score. **f**, DEGs between *Cgas*^*WT/WT*^ and *Cgas*^*WT*^^*/R241E*^ in IFN-MG and DAM-2-MG (FDR ≤ 0.05, log_2_[FC] ≥ 0.3). Oversized points represent genes linked with associated states (Supplementary Table 6). **g**, The relative proportions of microglial populations identified from snRNA-seq analysis of *Tmem119-creER*^*T2*^*-Cgas*^*WT/WT*^ (WT, *n* = 3) and *Tmem119-creER*^*T2*^*-Cgas*^*WT/R241E*^ (R241E, *n* = 2) microglia. **h**, Morris water maze test of *Tmem119-creER*^*T2*^*-Cgas*^*WT/WT*^ (*n* = 6) and *Tmem119-creER*^*T2*^*-Cgas*^*WT/R241E*^ (*n* = 11) mice. *P* = 1 × 10^−5^. **i**, Representative images and quantification of NeuN^+^ cells in the hippocampal CA1 region of *Tmem119-creER*^*T2*^*-Cgas*^*WT/WT*^ and *Tmem119-creER*^*T2*^*-Cgas*^*WT/R241E*^ mice. *n* = 5. Scale bars, 250 μm (left) and 50 μm (right). **j**,**k**, The relative survival of MAP2^+^ neurons cultured with *Rosa26-creER*^*T2*^*-Cgas*^*WT/R241E*^-isolated microglia treated with or without 4-OHT (*n* = 6) and with TNF-neutralizing (*n* = 4) or IFNAR-neutralizing antibodies (*n* = 3 slides), from *n* = 3 mice (**j**); or microglia from young and aged mice treated with TNF- or IFNAR-neutralizing antibodies (**k**) (averaged per mouse, *n* = 3). Data are mean ± s.e.m. *P* values were calculated using two-sided Student’s unpaired *t*-tests (**b**, **d**, **g** and **i**), one-way ANOVA followed by Tukey’s multiple-comparison test (**j** and **k**) and ordinary two-way ANOVA (**h**). Source Data

**Extended Data Fig. 8:**
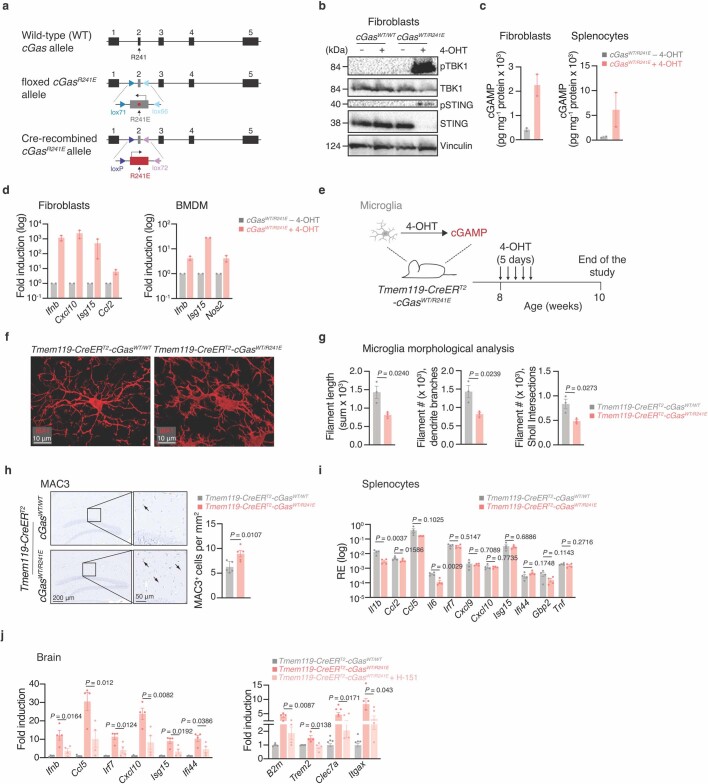
Genetic activation of cGAS-STING signalling in diverse cells of *cGas*^*R241E*^ mice in vitro and in vivo. **a**, Schematic illustrating the knock-in cGAS gain-of-function mouse model. **b**, Western blot analysis of cGAS activation in tail-tip fibroblasts of *Rosa26-creER*^*T2*^*-cGas*^*WT/WT*^ and *Rosa26-creER*^*T2*^*-cGas*^*WT/R241E*^ mice, treated or not with 4-OHT. **c**, cGAMP levels in cell lysates of tail-tip fibroblasts and splenocytes from *Rosa26-creER*^*T2*^*-cGas*^*WT/WT*^ and *Rosa26-creER*^*T2*^*-cGas*^*WT/R241E*^ mice (*n* = 2). **d**, mRNA expression levels of interferon-stimulated genes in tail-tip fibroblasts and bone-marrow-derived macrophages (BMDM) from *Rosa26-creER*^*T2*^*-cGas*^*WT/R241E*^ mice (*n* = 2), 4-OHT-treated or not. **e**, Experimental set-up for the analysis of microglia-specific cGAS activation. **f**, **g**, IBA1^+^ microglia analysis from *Tmem119-creER*^*T2*^*-cGas*^*WT/WT*^ and *Tmem119-creER*^*T2*^*-cGas*^*WT/R241E*^ mice by IMARIS, showing raw images pre-reconstruction (**f**) and morphological analysis (**g**), with image-based quantification (*n* = 3 mice). **h**, Representative images and quantification of hippocampal MAC3 in brain sections of *Tmem119-creER*^*T2*^*-cGas*^*WT/WT*^ or *Tmem119-creER*^*T2*^*-cGas*^*WT/R241E*^ mice (*n* = 5). **i**, mRNA expression levels of proinflammatory and interferon-stimulated genes in splenocytes of *Tmem119-creER*^*T2*^*-cGas*^*WT/WT*^ and *Tmem119-creER*^*T2*^*-cGas*^*WT/R241E*^ mice treated with 4-OHT (*n* = 4). **j**, Brain mRNA expression levels of proinflammatory and interferon-stimulated genes (left) and microglia activation markers (right) from *Tmem119-creER*^*T2*^*-cGas*^*WT/WT*^ and *Tmem119-creER*^*T2*^*-cGas*^*WT/R241E*^ mice 4-OHT-treated, and additionally with H-151 or not (*n* = 4). Data are mean ± s.e.m. *P* values were obtained with two-sided Student’s unpaired *t*-test (**g**-**i**) or one-way ANOVA followed by Tukey’s multiple comparisons tests (**j**). RE, relative expression. Source Data

Distinct immune signalling mechanisms have been implicated in neurodegenerative conditions^31^ that could synergise with the cGAS–STING signalling axis in promoting age-related neurodegeneration. To determine whether engagement of the cGAS–STING pathway alone is sufficient to drive neuropathological features, we devised a genetic strategy to trigger cGAS activity in vivo. Previously, we and others showed that cGAS is suppressed by binding to nucleosomes and that disruption of nucleosome regulation enables robust DNA-dependent activation of cGAS^32–37^ (Fig. 4a). We generated mice in which the nucleosome-binding mutant *Cgas*^*R241E*^ (corresponding to human *CGAS*^*R255E*^) can be conditionally expressed by Cre recombinase (Extended Data Fig. 8a). In vitro 4-hydroxytamoxifen (4-OHT) treatment of cells from mice with a tamoxifen-inducible *Rosa26-creER*^*T2*^ allele resulted in cGAMP synthesis and upregulation of type I IFN response genes, providing a proof of concept that *Cgas*^*R241E*^ mice can be used to study the effects of cGAS activation in vivo (Extended Data Fig. 8b–d).

We generated *Tmem119-creER*^*T2*^*-Cgas*^*WT/R241E*^ mice (hereafter microglial (mg)-*Cgas*^*R241E*^) allowing for tamoxifen-inducible expression of *Cgas*^*R241E*^ majorly restricted to microglia and brain macrophages (Extended Data Fig. 8e). At 2 weeks after administration of tamoxifen, mg*-Cgas*^*R241E*^ mice displayed a strong increase in the numbers of microglia with an activated morphology in several brain regions, including the hippocampus, compared with in the control mice (Fig. 4b,c and Extended Data Fig. 8f–h). Furthermore, the levels of inflammatory genes and type I IFN-related genes were elevated in the brains of tamoxifen-treated mg*-Cgas*^*R241E*^ mice (Fig. 4d). No elevation of inflammatory marker genes was detectable in peripheral organs, such as the spleen, ruling out a contribution of brain external processes to the observed changes (Extended Data Fig. 8i). As expected, H-151 treatment attenuated microglial activation in mg*-Cgas*^*R241E*^ mice (Extended Data Fig. 8j).

**Extended Data Fig. 9:**
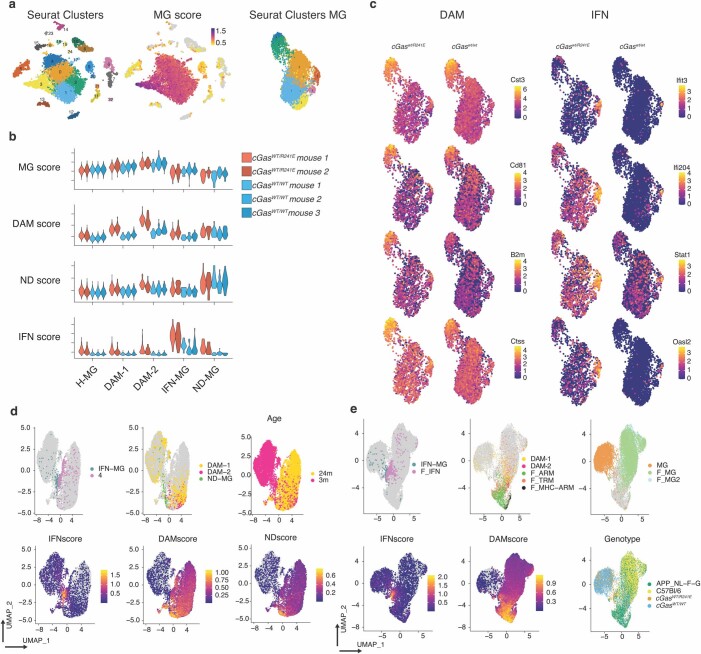
SnRNA-seq in microglia and hippocampi of mg-*cGas*^*R241E*^ mice. **a**, UMAP plots of unsupervised clustering of enriched microglia (25 individual clusters), microglia specific expression score, identifying clusters 0-4 and 17 as microglia, and unsupervised clustering of filtered microglia. **b**, Violin plots of gene expression scores related to microglia states between individual mice. **c**, Representative UMAP plots of genes associated with, and differentially expressed between *cGas*^*WT/R241E*^ and *cGas*^*WT/WT*^ mice in the DAM and IFN subpopulations. **d**, Integration of *cGas*^*WT/R241E*^ and *cGas*^*WT/WT*^ microglia with 24m aged microglia from (Tabula Muris, GSM4505405). UMAP plots of IFN-MG and Louvain cluster 4 (IFN-related aged microglia), DAM-1/2-MG and ND-MG, mouse age and gene expression scoring of microglia-related subpopulation genes showing co-clustering of IFN populations and DAM/ND populations with aged microglia expressing high levels of DAM and neurodegenerative (ND) markers. **e**, Integration of *cGas*^*WT/R241E*^ and *cGas*^*WT/WT*^ microglia with disease-associated microglia from (Sala Frigerio, GSE127892, F_IFN: Interferon response microglia, F_ARM: Activated Response Microglia, F_TRM: Transiting response microglia, F_MHC-ARM: Major Histocompatibility high ARMs, F_MG: homeostatic microglia). UMAP plots of IFN-MG and F_IFN, DAM-1/2-MGs and F_ARM, F_TRM, F_MHC-ARM, MG and F_MG/2 and gene expression scoring of microglia-related subpopulation genes showing co-clustering of IFN populations and DAM/TRM populations with disease-associated microglia expressing high levels of IFN and DAM markers.

**Extended Data Fig. 10:**
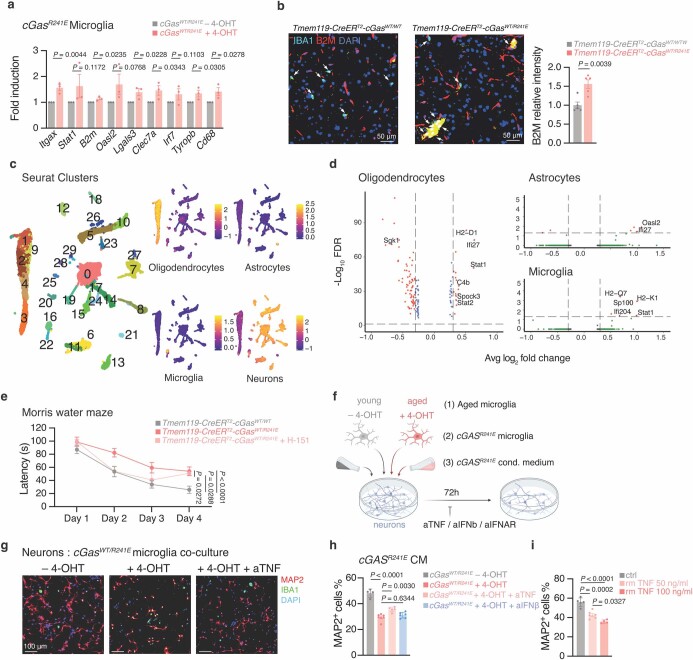
Effects of cGAS-activated microglia on brain cells. **a**, mRNA expression levels of interferon-stimulated genes and activation markers in microglia from *Tmem119-creER*^*T2*^*-cGas*^*WT/R241E*^ mice 4-OHT-treated or not (*n* = 3). **b**, Representative images and quantification of IBA1^+^ microglia B2M intensity from brain sections of *Tmem119-creER*^*T2*^*-cGas*^*WT/WT*^ and *Tmem119-creER*^*T2*^*-cGas*^*WT/R241E*^ mice (averaged per mouse, *n* = 5, from 10–15 cells). Arrows indicate IBA^+^ microglia. Scale bars, 50 μm. **c**, UMAP plots of sorted hippocampi nuclei’s unsupervised clustering (38 clusters) with identified oligodendrocytes-, astrocyte-, microglia- and neuron-specific gene expression scores. **d**, Volcano plots of genes differentially expressed between *Tmem119-creER*^*T2*^*-cGAS*^*WT/WT*^ and *Tmem119-creER*^*T2*^*-cGAS*^*WT/R241E*^ mice (*n* = 4) in oligodendrocytes, astrocyte, and microglia MG (FDR ≤ 0.05, Log2FC ≥ 0.3, genes listed in Supplementary Table 6). **e**, Morris water maze memory test of *Tmem119-creER*^*T2*^*-cGas*^*WT/WT*^ (*n* = 6) *and Tmem119-creER*^*T2*^*-cGas*^*WT/R241E*^ mice (data in Fig. 4h), additionally H-151-treated (*n* = 5) or not (*n* = 11), *P* = 1 × 10^−5^. **f**, Schematic illustrating neuronal cell co-culture experiments. **g**, Representative images of neurons (MAP2, red) cultured with microglia from *Rosa26-creER*^*T2*^*-cGas*^*WT/R241E*^ mice (IBA1, green), 4-OHT-treated or not, and aTNF (represents *n* = 3 experiments). Scale bars, 100 μm. **h**, Relative survival of MAP2^+^ neurons cultured with conditioned medium from *Rosa26-creER*^*T2*^*-cGas*^*WT/R241E*^-isolated macrophages, 4-OHT-treated (*n* = 6 FOV) or not (*n* = 4 FOV), and with aTNF (*n* = 5 FOV), aIFNb (*n* = 6 FOV, from *n* = 3 mice) addition, *P* = 3 × 10^−9^. **i**, Relative survival of MAP2^+^ neurons treated with increasing doses of recombinant TNF (untreated *n* = 5, 50 ng/ml *n* = 5, 100 ng/ml *n* = 4 FOV), from 3 slides per condition, *P* = 1 × 10^−5^. Data are mean ± s.e.m. *P* values were obtained by one-sided (**a**) or two-sided (**b**) unpaired Student’s *t*-test, one-way ANOVA followed by Tukey’s multiple comparisons tests (**h**,**i**) or two-way ANOVA (**e**). Source Data

To comprehensively define the cGAS-controlled transcriptional program of microglia, we performed single-nucleus RNA-seq (snRNA-seq) analysis of 9,505 cells enriched for microglia from which we bioinformatically identified 5,360 microglia obtained from tamoxifen-treated mg*-Cgas*^*R241E*^ mice and control *Cgas*^*WT/R241E*^ mice (Extended Data Fig. 9a). Clustering analysis revealed three distinctive transcriptional states of microglia displaying a unique transcriptional profile that segregated them from the main homeostatic microglial population (Fig. 4e and Extended Data Fig. 9b,c). These transcriptional clusters correspond to previously identified subsets, including disease-associated (DAM), IFN-associated and neurodegenerative microglial states that are associated with ageing and neurodegenerative conditions^38–41^ (Extended Data Fig. 9d,e). Cells from each of the three states strongly upregulated genes associated with the IFN signature (*Ifit*s, *Oasl1*, *Oasl2*, *Isg15*, *Stat2*), the DAM-signature (*B2m*, *Ctsb*, *Ctsd*, *Ctsz*, *Cd9*, *Cd63*) and the neurodegenerative signature (*Apbb2*, *Olfr111*, *Xylt1*)^38–40^ (Supplementary Table 3). Moreover, within the DAM subtype, we observed a progression from a lower to more highly activated state (DAM-1 to DAM-2), with a greater proportion of mg*-Cgas*^*R241E*^ cells present in the more activated state (Fig. 4e,g and Extended Data Fig. 9b). Comparison between the two mouse models revealed both an expansion and higher expression of genes associated with the IFN and DAM states (most notably within the DAM-2 population) in the mg*-Cgas*^*R241E*^ cells, and a general increase in IFN signature across the global microglial population (Fig. 4e,g, Extended Data Fig. 9b,c and Supplementary Table 4). We validated upregulation of type I IFN- and DAM-related genes in isolated microglia expressing *Cgas*^*R241E*^ and verified an increased proportion of B2M^+^ microglia in the brains of mg*-Cgas*^*R241E*^ mice (Extended Data Fig. 10a,b). Thus, these data establish that, in the absence of an additional trigger, cGAS alone is sufficient to promote transition of microglia to distinctive ageing- and disease-related activation states.

To resolve how cGAS activation in microglia affects other cell populations in the brain, we performed snRNA-seq analysis of the hippocampi of mg*-Cgas*^*R241E*^ mice that were treated or not with tamoxifen. Clustering analysis of 21,500 cells collected from four mice of each group revealed four major brain cell types, including microglia, astrocytes, oligodendrocytes and neurons (Extended Data Fig. 10c,d). Microglial cGAS activation was accompanied by IFN-related transcriptomic shifts in oligodendrocytes and astrocytes, whereas neurons displayed minimal transcriptional changes (Extended Data Fig. 10c,d and Supplementary Table 5). Among the genes that were most differentially expressed in oligodendrocytes were several genes that characterize this cell type during ageing and neurodegeneration, including *H2-D1*, *Ifi27*, *C4b*, *Spock3*, *Trf* or *Sgk1* (refs. ^42,43^). Thus, beyond cell-intrinsic effects in microglia, cGAS activation also directs transcriptional programmes in non-immune glial cells that resemble those occurring in aged brains.

## cGAS activation drives neurodegeneration

To determine whether cGAS activity in microglia can initiate neurodegenerative processes, we examined hippocampal-dependent learning and hippocampal neuron density as two independent measures for brain plasticity. Compared with the control group, mg*-Cgas*^*R241E*^ mice showed impaired cognitive performance in the Morris water maze test and concomitant loss of hippocampal neuron density (Fig. 4h,i). As in ageing, H-151 attenuated the deterioration of learning capacity in mg*-Cgas*^*R241E*^ mice, consistent with a model in which STING’s proinflammatory role affects neuronal function (Extended Data Fig. 10e). To dissect neurotoxic processes downstream of microglial cGAS–STING activity, we performed co-culture experiments with primary wild-type neurons and microglia from *Cgas*^*R241E*^ mice (Extended Data Fig. 10f). cGAS activity potently suppressed neuronal cell survival and this effect was also recapitulated using conditioned medium from *Cgas*^*R241E*^-expressing cells, indicating that cGAS-mediated neurotoxicity involves secretion of soluble mediators (Fig. 4j and Extended Data Fig. 10g,h). Analysis of our snRNA microglial transcriptome dataset revealed that *Tnf* is upregulated in the IFN-associated microglial state relative to homeostatic microglia and is induced in the brain of mg*-Cgas*^*R241E*^ mice (Fig. 4d and Supplementary Table 3). Notably, addition of a neutralizing antibodies against TNF led to strong rescue of neuronal death induced by *Cgas*^*R241E*^ (Fig. 4j and Extended Data Fig. 10h). By contrast, blocking type I IFN signalling had no effect (Fig. 4j and Extended Data Fig. 10h). Co-culture experiments with aged microglia confirmed a critical role for TNF in linking aberrant cGAS activity to compromised neuronal cell survival (Fig. 4k and Extended Data Fig. 10i).

## Discussion

Here we establish the cGAS–STING pathway as an important driver of ageing-related inflammation and define microglial intrinsic engagement of cGAS–STING in the establishment of ageing-associated neurodegeneration. The presence of a type I IFN signature in brain cells, in particular in microglia, is increasingly being associated with ageing and neurodegenerative disease in various species^13,15,39,44–50^. Our study in naturally aged mice demonstrates that activation of cGAS–STING signalling is an essential contributor to the ageing-related type I IFN response in microglia to direct neuronal loss and cognitive impairment. Single-cell transcriptional profiling of cGAS gain-of-function mice further reveals that, beyond type I IFNs, cGAS activation alone triggers a core microglial gene expression program that is shared between many neurodegenerative disease states and during ageing^38–40,51^. Thus, apart from natural ageing, these results point to a more extensive role for microglial cGAS–STING activation in degenerative brain disorders.

In vitro co-culture studies revealed that TNF is a critical neurotoxic factor of the cGAS-controlled microglial response. Furthermore, our snRNA-seq analyses uncovered considerable secondary changes in the activation states of oligodendrocytes and astrocytes, which can function as important intermediary cells in propagating neurotoxic signals. Elucidating the precise neuroimmune crosstalk that dictates microglial-dependent neurotoxicity downstream of cGAS will provide insights into their roles in neurodegeneration and is an important area for future investigations.

Our mechanistic characterization of activated microglia shows that mtDNA accumulates in the cytoplasm to stimulate aged cells, providing a functional link between two central features of ageing, namely mitochondrial dysfunction and inflammation. Previous research has revealed DNA damage products^5–7^ and retroelement amplification^52^ as upstream triggers of cGAS in senescence. We hypothesize that, in ageing, as in other complex diseases, distinct sources of DNA can act in synergy to promote cGAS activity. We propose that aberrant mtDNA also contributes to peripheral ageing-related inflammatory phenomena that are controlled by the cGAS–STING cascade.

Damage-associated triggers of the inflammatory response accompanying neurodegeneration may be generated in a disease-specific manner. Together with previous studies in models of Alzheimer’s disease^53^, Parkinson’s disease^54,55^, amyotrophic lateral sclerosis and frontotemporal dementia^56^, and Nieman–Pick’s disease^57^, our study reveals notable convergence on cGAS–STING signalling in chronic neurodegenerative conditions. On closer inspection, differences appear with regard to the molecular (genomic versus mtDNA) and cellular (microglia versus neurons) origin of immune activation, which will probably have repercussions on the characteristics of the disease itself.

## Methods

### Mice

Wild-type C57BL/6J mice and *Sting1*^*−/−*^ (STING-deficient) (025805) mice (aged 19–20 months) were purchased from The Jackson Laboratory. Mice were single-housed to prevent aggression-related injuries and fed standard chow diets. Aged mice were treated either with vehicle or 750 nmol H-151 in 200 µl PBS 5% Tween-80 as described in Fig. 1. In brief, aged mice were injected intraperitoneally with 750 nmol H-151 5 days a week for 8 weeks. After 8 weeks of treatment break, the mice were treated for another 8 weeks and then analysed using physical or cognitive functional tests. The mice were euthanized and tissues were collected for further analysis. Pharmacokinetic and tissue distribution experiments were performed by Pharmaron. *Sting1*^*−/−*^ mice and wild-type litters were bred and aged within the institute.

*Cgas*^*R241E*^ mice were generated using CRISPR–Cas9-mediated gene editing using a long single-strand oligo in C57BL/6J zygotes. The long single-stranded oligo contains exon 2 in reversed orientation and the R241E point mutation. This exon is flanked with lox71 and lox66 recombination sites in the opposite orientation and homology arms. The long single-strand was injected in mouse zygotes together with Cas9 protein (Integrated DNA Technologies) and two designed gRNAs (5′-TTTATAGGCACCCTATGTACAGG-3′ and 5′-CTGACCGCACGACTTACCCTGGG-3′) targeting the intronic region surrounding exon 2. *Cgas*^*R241E*^ mice were bred on B6;129*Gt(ROSA)26Sor*^*tm1(cre/ERT)Nat*^/J (The Jackson Laboratory, 004847) and C57BL/6-*Tmem119*^*em1(cre/ERT2)Gfng*^/J (The Jackson Laboratory, 031820) to generate *Rosa26-creER*^*T2*^*-Cgas*^*WT/R241E*^ mice and *Tmem119-creER*^*T2*^*-Cgas*^*WT/R241E*^ (mg-*Cgas*^*R241E*^) mice.

To induce microglial expression of *Cgas*^*R241E*^ in mg-*Cgas*^*R241E*^) mice, tamoxifen (75 mg per kg body weight) was intraperitoneally injected into 8–10-week-old mice once every 24 h on 5 consecutive days. Some mice also received 750 nmol H-151 daily, starting with tamoxifen (4-OHT, Sigma-Aldrich, T5648) injections. Within 1 week after the last administration of tamoxifen, some mice were analysed using cognitive tests and euthanized for tissue analysis.

To test the in vivo specificity of H-151, mice (aged 8–10 weeks) received 15 mg per kg of a STING agonist (DMXAA; InvivoGen) intraperitoneally. H-151 (750 nmol) was administered to one group 30 min before the injection of the STING agonist and, 3 h later, the mice were euthanized for tissue collection.

The cages were kept at 18–24 °C ambient temperature under 40–60% humidity. The mice were maintained under a 12 h–12 h light–dark cycle from 06:00 to 18:00. Food and water were available ad libitum. Mice were euthanized by anaesthetization by an injection of an overdose of sodium pentobarbital (150 mg per kg). Animal experiments were approved by the Service de la Consommation et des Affaires Vétérinaires of the canton of Vaud or obtained by IFM Therapeutics and were performed in accordance with the respective legal regulations.

### Morris water maze test

This test was performed to assess hippocampus-dependent spatial long-term memory and learning. The Morris water maze consists of a circular tank filled with water containing a non-toxic coloured paint to contrast with the mouse fur, and maintained at room temperature (23 ± 1 °C). To escape the water, the mice had to find a platform. This escape platform was submerged and was not visible for the animals. The tank was placed in a room with constant extra-maze visual cues and ambient light throughout all the experiment. Mice were trained during a maximum of 5 days to find the escape platform using visual cues as spatial points of reference. Each day, each mouse went through a maximum of 6 trials. The maximum duration of each trial was 120 s. At the end of each trial, the mouse was placed back into its home cage under a heating lamp for recovery and resting time.

### Fear conditioning test

On each day of the experiment, the animals were moved to the experimental room and left undisturbed for 30 min in their home cage. On the first day, the mice were placed into a conditioning chamber, and a tone was presented followed by a foot shock. The tone–foot-shock pairing was repeated a maximum of four times during the training session. On the second day, the mice were re-exposed to the context with no tone, and the freezing response was monitored. Mice only received a foot shock on the first day of the experiment and the foot shock lasted a maximum of 2 s with a maximum intensity of 0.5 mA.

### Forelimb grip strength test

The assessment of limb strength in aged mice was performed using a commercially available automatic grip strength meter. This test is based on the natural tendency of the mouse to grasp a bar or a grid when it is suspended by the tail and gently pulled backwards. The peak resistance force after the mouse lost grip from the grid was recorded as it was being pulled away from the device.

### Treadmill stress test

For the exercise test, the mice were placed on a treadmill. Plexiglas walls limit the treadmill and, next to the running lane, an electric grid dispenses small shocks when mice touch it to force them to run. Mice were habituated to the treadmill for 5 min before the test. Mice started running at a very moderate speed (15 cm s^−1^). The speed gradually increased at 3 cm s^−1^ every 12th minute. The device automatically monitored the run distance and the number of shocks received or the time of air-puff stimulation. When a mouse received more than five shocks in two consecutive minutes, it was considered that the mouse reached exhaustion and the mouse was placed back into its home cage with full access to food and water. The shocks (0.1 mA) were of very low intensity, enough to cause an unpleasing feeling (like a sting) to keep them running and far from resulting in damage to their integrity.

### Whole-mount immunostaining

We performed whole-mount immunostaining followed by confocal microscopy to examine the infiltration of F4/80^+^ macrophages in adipose tissue. The mice were perfused with PBS before the collection of inguinal white adipose tissue. The tissue depots were subdivided into 0.5–0.75 cm^3^ sized pieces, fixed in 1% paraformaldehyde (PFA) for 30 min at room temperature with gentle rocking, and washed in PBS three times for 10 min under gentle rocking. The tissue was blocked for 30 min in 5% BSA in PBS, then incubated with primary anti-F4/80 antibodies (Thermo Fisher Scientific, A3-1, MA1-91124) diluted 1:100 in blocking buffer overnight at 4 °C. The samples were washed with times with PBS before incubation with secondary anti-rat-Alexa 488 antibodies (Thermo Fisher Scientific, A-21208) diluted 1:350 in blocking buffer for 1 h at room temperature with gentle rocking. After three washes with PBS, the samples were incubated for 20 min in a lipid- and nucleus-staining cocktail composed of 1 µg ml^−1^ DAPI (Sigma-Aldrich, D9542) and 0.25 µg ml^−1^ BODIPY (Thermo Fisher Scientific, D3922) and washed three times in PBS before imaging. The images were taken on an inverted scanning confocal microscope (Zeiss LSM 700 Inverted) and assembly of 3D reconstructions was accomplished by taking *z*-stack images.

### Immunofluorescence and immunohistochemistry analysis

For mouse tissue experiments, formalin-fixed paraffin-embedded mouse tissue blocks were cut into 3 μm sections for immunohistochemistry staining and 6 μm sections for immunofluorescence staining and placed onto slides. The sections were first deparaffinized and rehydrated, then heat-induced epitope retrieval was performed and the sections were permeabilized with PBS 1% (v/v) Triton X-100. The samples were stained with primary antibodies (IBA1, Abcam, ab178846, rabbit, 1:10,000; IBA1, Abcam, ab5076, goat, 1:300; Mac3, Becton Dickinson, 553322, 1:150; GFAP, Agilent, Z033429-2(AGI), 1:100; NeuN, Merck, MAB377, 1:100; synaptophysin, Cell Signaling, 36406S, 1:500; and B2M, Abcam, ab75853, 1:100) overnight at 4 °C. For immunofluorescence analysis, the sections were then stained with fluorescently labelled secondary antibodies (anti-rabbit-AF488 (A-21206, 1:300), anti-rabbit-AF568 (A-10042, 1:250), anti-goat-AF488 (A-11055, 1:200), all from Thermo Fisher Scientific) for 90 min at room temperature. For immunohistochemistry analysis, the sections were stained with HRP-conjugated secondary antibodies (anti-rabbit-HRP, 711035152, 1:300; and anti-mouse-HRP, 715035150, 1:300; both from Jackson ImmunoResearch) followed by DAB staining and Harris counterstaining. Images from immunohistochemistry staining were acquired using either a Zeiss AxioScan 7 slide scanner or Olympus VS120 whole-slide scanner and collected using the Olympus OlyVIA software. Data were manual counts of parenchymal IBA1^+^ microglial cells, Mac3^+^ cells and NeuN^+^ cells. Cell counts were normalized to the hippocampal area. Images from immunofluorescence staining were analysed using wide-field fluorescence microscope (Zeiss Axioplan) and processed using ImageJ.

Microglia isolated from young and aged mice were imaged using the Leica SP8 confocal microscope with a white light laser. Slices or microglia were imaged with a 63× oil-immersion objective (NA = 1.45, Leica), with the standard settings across samples labelled the same way. *z*-Stacks were subsequently deconvolved using the Huygens Deconvolution Software (Scientific Volume Imaging), and 3D views were rendered using IMARIS (Bitplane). The following parameters were used for the generation of the surfaces in IMARIS for the visualization of pSTING and IBA1. Hippocampal slices from the aged mouse in Extended Data Fig. 6b, pSTING: manual threshold: 90, smoothing: 0.141 μm. Hippocampal slices from the aged mouse in Extended Data Fig. 6b, IBA1: manual threshold: 25.7, smoothing: 0.5 μm. Isolated microglia from young and aged mouse brains in Extended Data Fig. 6c, pSTING: manual threshold: 110, smoothing: 0.120 μm.

For quantitative analysis of the proportion of pSTING^+^ and IBA1^+^ cells, maximum intensity projection using the s.d. projection type was rendered using ImageJ. A cut-off intensity threshold of 32 was applied to the pSTING channel, and the number of pSTING^+^ cells and IBA1^+^ cells and the total number of cells in the field of view was enumerated.

For IMARIS images of microglia, morphometrical analysis of microglial cells was conducted as previously described^58^. In brief, the animals were perfused with ice-cold PBS. The brains were fixed in 4% PFA overnight. The tissue was then dehydrated in 30% sucrose and embedded in Tissue Tek (Sakura). Cryostat sections (thickness, 50 µm) were blocked in 5% BSA supplemented with 0.5% (v/v) Triton X-100 in PBS. The sections were then incubated with anti-IBA1 primary antibodies (Abcam, ab178846, 1:1,000) for three nights. Donkey anti-rabbit Alexa Flour 568 antibodies (Thermo Fisher Scientific, A10042, 1:500) were used as the secondary antibodies and were applied for one night. Nuclei were visualized using DAPI. Imaging was performed using the SP8 confocal microscope (Leica) and a 63× objective (step size, 0.4 µm). The cells were visualized in 3D and reconstructed using IMARIS (v.9.6, Bitplane).

For neuronal culture staining, cell culture coverslips were fixed in 4% PFA for 10 min, washed twice with PBS, then permeabilized in 2% saponin, 0.1% (v/v) Triton X-100 in PBS for 15 min at room temperature, and blocked in PBS with 1% BSA for 1 h at room temperature. The coverslips were incubated overnight at 4 °C with primary antibodies against MAP2 (Sigma-Aldrich, M4403, 1:250) and IBA1 (Abcam ab178846, 1:250). The samples were washed with PBS and incubated with secondary antibodies (anti-rabbit-AF568 (A-10042), anti-mouse-AF488 (A-11001), both 1:250) for 90 min at room temperature. Cell nuclei were stained by incubating with 5 µg ml^−1^ DAPI for 10 min, and the coverslips were mounted with fluorescent mounting medium (Agilent, S302380). Images were acquired using a wide-field fluorescence microscope (Zeiss Axioplan). The same settings were applied to all images within the same experiments. All analyses were performed using ImageJ. A list of all of the antibodies used is provided in Supplementary Table 2.

### Human adipose tissue explants

All material used in this study was obtained from the Cohort of Obese Patients of Lausanne with an ethically approved license by the commission of Vaud Canton (CER-VD project PB_2018-00119). The coded samples were collected under signed informed consent conforming to the guidelines of the 2000 Helsinki declaration. Human adipose tissue was resected during gastric bypass surgery from participants with obesity. Three of the participants were male, and eight of the participants were female. The mean age of the participants was 48.7 years old; s.d., 9.7; range, 30–60. No participant was known to have a malignancy. Greater omental adipose tissue was obtained from each participant. Adipose tissue was cut into small pieces, washed with PBS three times and cultured in medium containing 1 mM sodium pyruvate (BioConcept), 2 mM glutamine (Life Technologies), MEM vitamins (Life Technologies), MEM non-essential amino acids (Thermo Fisher Scientific) and antibiotics. The explants were treated with H-151 (daily, 1–2 µM), DMSO, or with 20 µM quercetin and 1 µM dasatinib (Chemie Brunschwig). After incubation for 6 days, part of the adipose tissue was fixed before SA-β-galactosidase staining (with or without tissue clearance), and the remaining—if sufficient—was collected for RNA isolation. The conditioned medium was collected (in three to five replicates per patient) for ELISA analysis of IL-6, IL-8 and MCP-1.

### Adipose tissue clearance

The adipose tissue from patients was immersed in 4% formalin (Sigma-Aldrich) for 2 days on a rotary shaker at 4 °C, then washed for 4 days on a rotary shaker in PBS at 4 °C. The tissue was washed three times for 30 min in PBS on an orbital shaker at room temperature, then sectioned to 500 µm slices with a vibratome. The sample was permeabilized in 2% PBS-T (2% Triton X-100 in PBS solution containing 0.05% sodium azide) for 2 days on an orbital shaker at room temperature, then cleared in RapiClear (RapiClear 1.52, SUNJin Lab, RC152001) overnight at room temperature. The cleared tissue was mounted in fresh RapiClear reagent and imaged using a confocal microscope (Zeiss LSM 700 Inverted).

### SA-β-Gal assay

Both cells and tissue explants were washed twice with PBS, then fixed for 5 min with 2% formaldehyde (Sigma-Aldrich) and 0.2% glutaraldehyde (Sigma-Aldrich) in PBS at room temperature, washed with PBS and then incubated overnight at 37 °C in staining solution with 40 mM citric acid NA phosphate, 5 mM K_4_Fe(CN)_6_, 5 mM K_3_Fe(CN)_6_ (Fluka analytical, 34272), 150 mM NaCl, 2 mM MgCl_2_ and 1 mg ml^−1^ X-Gal (Roche, R0404) in water. The samples were washed twice with PBS before imaging by microscopy (Zeiss Axio Vert.A1).

### RNA extraction

Human and mouse tissues were snap-frozen in liquid nitrogen and stored at −80 °C until processing. RNA was isolated using the TRIzol–chloroform (Thermo Fisher Scientific, 15596018) method and a tissue homogenizer (Thermo Fisher Scientific). For cells, RNA was isolated using the RNeasy Mini Kit (Qiagen, 74004) (cell lines) or the RNeasy Micro Kit (Qiagen, 74104) (primary microglia), according to the manufacturer’s instructions.

### Bulk RNA-seq

RNA was isolated from the control and irradiated WI-38 cells, treated daily or not from day 10 to day 20 with STING inhibitor H-151 (0.5 µM) using the RNeasy Mini kit (Qiagen, 74004). RNA was further processed for sequencing by the Gene Expression Core Facility GECF at EPFL. mRNA-seq libraries were prepared using the TruSeq mRNA stranded LT (Illumina kit). The samples were sequenced using the NextSeq 500 system sequencing with 1 × 75 cycle (single read), high output mode (expected a minimum of 400 million reads), chemistry v2. Sequencing data were processed using HTSstation online software from the Bioinformatics and Biostatistics Core Facility at EPFL. Heat maps were produced from normalized expression data using Cluster 3.0 for computation and JTreeview for visualization.

RNA isolated from mouse brains was processed as follows: RNA quality was controlled on the TapeStation 4200 (Agilent), confirming that all were of good quality (scores > 8.1). Libraries for mRNA-seq were prepared using the stranded mRNA ligation method (Illumina) starting from 800 ng RNA, according to the manufacturer’s instructions. Libraries, all bearing unique dual indexes, were subsequently loaded onto the NovaSeq 6000 flow cell (Illumina) and sequenced according to the manufacturer instructions, yielding for each sample at least 40 million pairs of 60-nucleotide-long reads. Reads were trimmed of their adapters using BCL Convert (v.3.9.3; Illumina) and quality-controlled using fastQC (v.0.11.9).

### Bulk RNA analysis

For the fibroblasts, RNA was mapped to the human genome assembly hg38 (gencode v36, Ensembl 102) using STAR aligner (v.2.7), and counts were generated with HTSeq Count. In total, 13,006 protein-coding genes (cpm > 1 in at least 2 samples) were retained for analysis. Differential gene expression analysis was performed in R (v.4.0) using voom-Limma (v.3.28).

For bulk hippocampus, RNA was mapped to the mouse genome assembly GRCm39 (release 109) using STAR aligner (v.2.7.10b), and counts were generated using featureCounts (v.2.0.1). DEG analysis was performed using the R (v.4.2.2) package DESeq2 (v.1.38.2).

### scRNA-seq

Nuclei from mouse brain were extracted by homogenizing mouse brain tissues in Nuclei EZ Lysis Buffer (Millipore Sigma) using a douncer. Nuclei were labelled with DAPI (10 µg ml^−1^, Sigma-Aldrich) and sorted for sequencing. To enrich microglial cells, in addition to DAPI staining, nuclei were stained with anti-RBFOX3/NeuN-647 (Novus Biologicals) and anti-Olig2-488 (Merck), and DAPI^+^NeuN^−^Olig2^−^ cells were sorted for analysis. The gating strategy used for microglial nucleus sorting is depicted in Supplementary Fig. 2. Sorted mouse brain cell nuclei were washed and resuspended in PBS 1% BSA supplemented with 0.2 U µl µg^−1^ RNase inhibitor, checked for the absence of significant doublets or aggregates and loaded into a Chromium Single Cell Controller (10x Genomics) in a chip together with beads, reverse transcription master mix reagents and oil to generate single-cell-containing droplets. Single-cell gene expression libraries were then prepared using the Chromium Single Cell 3′ Library & Gel Bead Kit v3.1 (PN-1000268) according to the manufacturer’s instructions (protocol CG000315 Rev C). Quality control was performed using the TapeStation 4200 (Agilent) and the QuBit dsDNA high sensitivity assay (Thermo Fisher Scientific) according to the manufacturer’s instructions. The sequencing libraries were loaded onto an Illumina NovaSeq Flow Cell and sequenced using read lengths of 28 nucleotides for read1 and 90 nucleotides for read2, at a depth of around 80,000 reads per cell. Illumina BCL convert was used to demultiplex reads, after which 10x Genomics Cell Ranger Single Cell Software Suite (v.7.0.0) was used to perform barcode processing and 3′ gene counting using 10x Genomics custom annotation of mouse genome assembly mm10.

### Single-cell analysis

For the microglial and hippocampus datasets, the median sequencing depth was 107,267 and 100,912 reads per cell, with 91.5% and 94.6% of the reads confidently mapping to the genome and 9,748 and 21,585 nuclei captured, respectively. Sample processing and analysis were performed using the R package Seurat^59^ (v.4.1.1.9003). For each dataset, we filtered out low-quality and potential doublet cells on the basis of violin plot distributions of genes, UMI counts and mitochondrial gene expression (as determined using the Seurat PercentageFeature function) per cell. Filters were set on a per-experiment basis as sequencing run and cell type significantly influence filtering cut-offs. For both single-nucleus microglial and whole-hippocampus datasets, only genes detected in a minimum of three cells, and cells with a minimum of 200 genes were included. For single microglia, cells with ≥5% mitochondrial gene expression, ≥4,500 genes and ≥10,000 UMIs were excluded and, in single hippocampi, cells with ≥5% mitochondrial gene expression, ≥8,000 genes and ≥45,000 UMIs were excluded. After filtering, we retained 5,360 (1,001, 514, 924, 1,468 and 1,453 per sample, 1:2 *Cgas*^*WT/R241E*^, 3:5 *Cgas*^*WT/WT*^) and 21,500 (2,076, 2,655 2,383, 2,769, 3,550, 3,422, 2,547 and 2,098 per sample, 1:4 *Cgas*^*WT/R241E*^, 5:8 *Cgas*^*WT/WT*^) nuclei per dataset, respectively. After merging, the samples were evaluated for potential batch effects. As we observed individual sample-based cell type cluster offsetting, probably a result of mean expression differences, we performed the Seurat Integration^60^ preprocessing workflow with a SCTransform (v2) normalization (negative binomial model) for cell clustering^61^, both for the microglial and hippocampus datasets. Integration was performed on the basis of 3,000 anchor genes, and subsequent reductions in dimensionality were performed on the basis of the top 30 principal components. Unsupervised clustering was performed at a resolution of 1 and 0.8 for the microglia and hippocampi, respectively.

Cluster identity was determined on the basis of known cell type marker genes and cross-referenced to The Human Protein Atlas single-cell reference data (https://www.proteinatlas.org/) (Supplementary Table 6). Cell identity gene expression scores were assigned to individual cells on the basis of the overall expression of given gene lists using the AddModuleScore function (based on a function of Seurat^62^) (Extended Data Fig. 9a,f) and visualized using the FeaturePlot_scCustom function from the R package scCustomize (v.0.7.0), allowing for subsequent analysis of specific cell types (such as microglia, oligodendrocytes) (Supplementary Table 5). In the microglial dataset, contaminating non-microglial cells were filtered out on the basis of the microglial score, and the remaining cells were further subsetted and reclustered, as described above (Extended Data Fig. 9a). Particular attention was given to the removal of MRC1^+^ macrophages, which often display a similar expression profile to microglia, and can be an unwanted source of variation. Unsupervised clustering was performed at a resolution of 0.8 for the subsetted dataset. After subletting, an average of 1,571 and 3,026 genes, 2,762 and 9,112 UMIs, and 0.069% and 0.028% mitochondrial gene content were found per nuclei in the microglial and hippocampal datasets, respectively. To identity subtypes of microglia, we used the same method as described above, using the Seurat AddModuleScore function for gene lists related to IFN, DAM, neurodegenerative and H-MG (Supplementary Table 6—gene sets were derived from refs. ^38,39^, Fig. 4e and Extended Data Fig. 9b). After cluster cell type identification for both datasets, differential gene expression analysis was performed using the FindMarkers function of Seurat, using the MAST test (v.1.22.0)^63^, which implements a two-part hurdle model, with a Bonferroni correction. Data were normalized using the NormalizeData (LogNormalize) function in Seurat before differential gene analysis. Microglial cluster identity DEGs for DAM, IFN and neurodegenerative-associated microglia were calculated with the H-MG used as a reference.

For the comparison to the aged^13^ and disease-associated microglia^40^, raw datasets and metadata were acquired from the Gene Expression Omnibus (GEO: GSM4505405 and GSE127892, respectively). Data were processed using R and the Seurat package. For the aged dataset, raw counts were extracted for 24-month-old microglia only, and merged with the relevant metadata, including previously identified Louvain clusters. In the case of the disease-associated microglial dataset, no cluster annotations were readily available; as such, the analysis was recreated from the raw counts using the materials and methods of the published article. We identified all relevant clusters from the previous article, as well as an additional subcluster of ARMs that showed high expression of MHC class I genes (*H2-Aa*, *H2-Ab1*, *H2-Eb1*), representative of an activated microglial state, which we labelled F_MHC-ARM. We individually integrated these datasets with our final annotated microglial dataset using Seurat’s standard integration workflow with 2,000 variable features, 11 PCA dimensions for clustering, and a clustering resolution of 0.5 and 0.3 for the aged and disease datasets, respectively. After integration, we used the AddModuleScore function as described above to assign an IFN, DAM and neurodegenerative score and compared clustering of the previously annotated cell types between datasets.

### RT–qPCR analysis

For mouse organ tissue, cells and human patient tissue, RNA was reverse-transcribed using the RevertAid First Strand cDNA Synthesis reagents (Thermo Fisher Scientific), and RT–qPCR was performed in duplicates or triplicates using the Maxima SYBR Green Master Mix (Thermo Fisher Scientific) on QuantStudio 6/7 qPCR instruments. A list of the primer sequences used is provided in Supplementary Table 1.

### Immunoblotting

Cell pellets were lysed in a lysis buffer containing 10 mM Tris (pH 8), 1 mM EDTA, 0.5 mM EGTA, 1% (v/v) Triton X-100, 0.1% sodium deoxycholate, 0.1% SDS, 140 mM NaCl, protease and phosphatase inhibitors (protease inhibitor cocktail I, animal-free, CALBIOCHEM). Protein concentration was measured using the BCA Pierce Protein assay kit (Thermo Fisher Scientific) and normalized to the lowest concentration. The primary antibody was incubated in 2.5% milk or BSA in PBS-T overnight at 4 °C. The secondary anti-mouse or anti-rabbit HRP-conjugated antibodies were incubated for 1 h at room temperature. Proteins were visualized with the enhanced chemiluminescence substrate ECL (Pierce, Thermo Fisher Scientific) and imaged using the ChemiDoc XRS BioRad Imager and Image Lab Software. Imaging was performed in two channels: chemiluminescence and colorimetry. Uncropped images are provided in Supplementary Fig. 1. A list of all of the antibodies used is provided in Supplementary Table 2.

### Primary cell isolation

Splenocytes: to isolate mouse splenocytes, the spleen was mashed through a 70 µm cell strainer using the plunger end of a syringe, cells were resuspended in medium and centrifuged at 800*g* for 3 min. Red-blood cells were lysed in 2 ml ACK lysing buffer (Life Technologies, A1049201) for 5 min at room temperature, inactivated by 20 ml complete medium, centrifuged at 800*g* for 3 min and washed once more with PBS. The pellet was kept at −80 °C until further analysis.

Tail-tip fibroblasts: a 2 cm portion of the mouse tail was cut, incubated in ethanol 70% for 5 min and air dried. The tissue was minced with scissors and incubated with digestion buffer containing 2.5 mg ml^−1^ collagenase D (Sigma-Aldrich, 11088866001) and 1.25 mg ml^−1^ Pronase (Millipore, 53702) in complete medium for 90 min at 37 °C, shaking at 200 rpm. Single cells were isolated by filtering through a 70 µm cell strainer, centrifuged at 580*g* for 7 min, resuspended in 10 ml medium and plated into a 10 cm dish.

Microglia: The whole brain from adult mice was removed and minced into small pieces. The brain pieces were further dissociated using the Adult Brain Dissociation Kit (Miltenyi Biotec, 130-092-628) according to the manufacturer’s instructions. Dissociated cells were filtered through a 100 µm cell strainer. The debris was removed by gradient centrifugation using the Debris Removal Kit (Miltenyi Biotec, 130-109-398). The cells were labelled with anti-CD11b-beads (Miltenyi Biotec, 130-093-634) and positively selected by magnetic sorting.

### Cell culture

Human fibroblast cells (BJ, WI-38) were cultured under 5% CO_2_ and 5% O_2_ at 37 °C in Dulbecco’s modified Eagle medium (DMEM) (Life Technologies) containing 10% (v/v) FCS, 1% (v/v) penicillin (100 IU ml^−1^)–streptomycin (100 μg ml^−1^). Mouse tail-tip fibroblasts were cultured under 5% CO_2_ and 20% O_2_ at 37 °C. Mouse bone-marrow-derived macrophages were generated using L929-cell-conditioned medium as a source of granulocyte/macrophage colony-stimulating factor. Mouse cells (BV2) were cultured under 5% CO_2_ and 20% O_2_ at 37 °C in minimum essential medium (MEM) GlutaMAX Supplement (Gibco, 41090036) containing 10% (v/v) FCS, 1% (v/v) penicillin (100 IU ml^−1^)–streptomycin (100 μg ml^−1^). Cells were repeatedly tested for mycoplasma using specific primers. Cell lines were purchased from ATCC (BJ, CRL-4001, WI-38, CCL-75) and amsbio (BV-2, AMS.CL-0493-1).

### Primary microglial culture

Isolated microglia were cultured in DMEM (Life Technologies) containing 10% (v/v) FCS, 1% (v/v) penicillin (100 IU ml^−1^)–streptomycin (100 μg ml^−1^), GlutaMAX (Thermo Fisher Scientific, 31331028), GM-CSF (50  ng ml^−1^, ImmunoTools, 12343123) and M-CSF (100 ng ml^−1^, ImmunoTools, 12343112). Cells were treated with H-151 (0.5 μM daily) or VBIT-4 (10 μM every other day, Selleckchem, S3544) for 4 days before gene expression analysis. The relative survival of primary microglia treated or not with H-151 (1 μM) was assessed 24 h after treatment using the CellTiter-Blue Cell Viability Assay (Promega, G808A), according to the manufacturer’s instructions.

### Generation of *Cgas*-KO BV2 cells

sgRNA (5′-ATATTCTTGTAGCTCAATCC-3′) was cloned into px458-GFP vector (Addgene, 48138). BV2 cells were transfected with the generated vector. Then, 2 days after the transfection, live GFP-positive single cells were sorted into 96-well plates. Growing cells were tested for deletion using immunoblot analysis.

### Neuronal cultures

Primary mouse cortical neuron cultures derived from wild-type mice were cultured on 35-mm-well plates (5 × 10^5^ cells per well) coated with Cultrex poly-lysine (Trevigen) in medium consisting of Neurobasal (Invitrogen), B27 supplement (Invitrogen), l-glutamine (Invitrogen) and penicillin–streptomycin (Invitrogen). For young/aged microglial co-culture experiments, primary microglia isolated from young (8 to 12 weeks old) and aged (24 to 27 months olds) mice were added 1:1 onto hippocampal neurons, 4 h after they were seeded onto coverslips, and additionally treated with anti-TNF (Bio X Cell, XT3.11, BE0058, 25 μg ml^−1^) or anti-IFNAR (Sigma-Aldrich, MARI-5A3, 10 μg ml^−1^) neutralizing antibodies. For microglia *Cgas*^*R241E*^ co-culture experiments, primary microglia isolated from *Rosa26-creER*^*T2*^*-Cgas*^*WT/R241E*^ mice were added 1:2 onto cortical neurons, 4 h after they were seeded onto coverslips, and 4-OHT (Sigma-Aldrich, T176, 600 μM) and mouse anti-TNF neutralizing antibodies (Bio X Cell, clone XT3.11, BE0058, 25 μg ml^−1^) or anti-IFNAR neutralizing antibodies (Sigma-Aldrich, MARI-5A3, 10 μg ml^−1^) were added or not to the medium and the cells were co-cultured for 72 h. For conditioned medium experiments, macrophages were isolated from *Rosa26-creER*^*T2*^*-Cgas*^*WT/R241E*^ mice and differentiated in the presence or absence of 4-OHT (600 μM). Their conditioned medium (from day 5 to day 8) was collected, filtered through a 22 μm mesh and added (diluted 1:2 in neurobasal medium) onto neuronal cultures for 72 h in addition or not to mouse anti-TNF (Bio X Cell, XT3.11, BE0058, 25 μg ml^−1^) or anti-IFNβ (R&D, MAB8234, 10 μg ml^−1^) neutralizing antibodies. For the analysis of co-culture experiments, counts were averaged per slide from 4 to 5 fields of view, obtained from 3 mice (*Rosa26-creER*^*T2*^*-Cgas*^*WT/R241E*^ microglia) or averaged per mouse (*n* = 3), obtained from 3 to 4 fields of view (aged microglia).

### Click-iT EdU-incorporation assay

The Click-iT EdU reaction was performed according to the instructions of the Click-iT imaging kit (molecular probes by Life Technologies). Four images per condition were acquired using a ×40 magnification objective. The number of EdU-positive cells and DAPI-positive cells was determined using ImageJ.

### Senescence induction and STING inhibitor treatments

Human fibroblasts (WI-38 and BJ) were either irradiated with 12 Gy (RS-2000 X-Ray Irradiator), treated with 1 μM abemaciclib (Selleckchem, S7158) or with 5 μg ml^−1^ bleomycin (Chemie Brunschwig, TRCB595750). The medium was changed every 72 h and, after 10 days, the cells were treated daily with STING inhibitor H-151 for 10 more days (0.5 µM). To obtain replicative senescent cells, WI-38 cells were serially propagated (at each passage, after reaching 80% confluence, cells were trypsinized and diluted 1:4) until proliferation ceased (approximately 90 days), and were then treated daily with STING inhibitor H-151 (0.5 µM) for 1 to 3 weeks. Mouse BV2 microglial cells were irradiated with 10 Gy (RS-2000 X-Ray Irradiator), the medium was exchanged every other day and the cells were treated daily with DMSO or H-151 (1 µM) from day 4 to day 6 after irradiation.

### Generation of mtDNA-depleted ρ^0^ microglia

BV2 cells were pretreated with 2′,3′-didoxycytidine (ddC, Sigma-Aldrich, D5782) for 6 days (20 µM, renewed every other day) before irradiation (10 Gy), the medium was exchanged every other day and the cells were collected for analysis on day 6. To verify the integrity of ρ^0^ BV2 cells, they were stimulated with LPS (from *Escherichia coli* serotype EH100(ra), Enzo, 100 ng ml^−1^) or dsDNA 90-mer transfection (2 μg per well of a 6-well plate, using Lipofectamine 2000 reagent, Thermo Fisher Scientific, according to the manufacturer’s instruction) and collected 3 h after.

### mtDNA extraction and quantification

For whole-cell mtDNA analysis, control and ddC-treated BV2 cells were collected on day 6. Total DNA was purified using the DNeasy Blood and Tissue Mini Kit (Qiagen, 69504) according to the manufacturer’s instructions. For analysis of the cytosolic fraction in BV2 cells, DNA was isolated from either control or irradiated cells (wild-type and ρ^0^, collected on day 6 after irradiation) using the Mitochondria/Cytosol Fractionation Kit (BioVision, K256) according to the manufacturer’s instructions. For the analysis in young and aged mouse primary microglia, cells were fractionated using digitonin isolation buffer (150 mM NaCl, 50 mM HEPES, 25 μg ml^−1^ digitonin) for 10 min on ice, then centrifuged at 2,000*g* for 5 min to separate cytosolic and membranous extracts. DNA was isolated from all fractions using the DNeasy Blood and Tissue Mini Kit (Qiagen, 69504) according to the manufacturer’s instructions. Isolated DNA (diluted to 20 ng ml^−1^) was used as a template for qPCR analysis of mitochondrial DNA sequences *Mito* and *CoI* expression, using *B2m* as a nuclear control.

### Intracellular DNA imaging

Super-resolution Airyscan images were acquired on a Zeiss LSM 980 with Airyscan microscope (Carl Zeiss). Samples were prepared on the CellCarrier 96 ultral microplate (PerkinElmer). TOMM20 and dsDNA were labelled with monoclonal rabbit antibodies against TOMM20 (Abcam, ab232589, 1:500) and monoclonal mouse antibodies against dsDNA (Sigma-Aldrich, MAB1293, 1:500), respectively. The samples were then washed and incubated with Alexa Fluor 568/488 secondary antibodies. The nucleus was stained with Hoechst 33342 (Sigma-Aldrich, B2261). Data were collected using a ×63/1.4 NA objective for the majority of experiments, under the control of the Zeiss ZEN software; 405, 488 and 561 nm laser lines were used. In cases in which *z*-stacks were collected, the software-recommended optimal slice sizes were used. Airyscan image processing was performed using the Airyscan processing function with the standard mode in the ZEN software. To maintain clarity and uniformity throughout the paper, the images were pseudocoloured.

### 3D renderings, DNA quantification and image analysis

Acquired *z*-stacks were imported into IMARIS (Bitplane). For cytosolic DNA focus quantification inside and outside the mitochondrial outer mitochondrial, a surface was created using the TOMM20 channel and spots were generated from the dsDNA channel. The shortest distances between spots to surfaces were obtained and analysed to distinguish dsDNA inside or outside mitochondrial outer mitochondrial. Distances more than 1 μm were excluded. Distances with positive or negative values were defined as outside or inside the mitochondrial outer mitochondrial. Images were analysed using IMARIS and ImageJ, including the snapshot images in 3D-view mode.

### Transfection

Human BJ fibroblasts were irradiated (12 Gy) and, when fully senescent (day 10), were transfected with siSTING or siNC (20 ng of RNA per 6 well) using Lipofectamine 2000 reagent (Thermo Fisher Scientific) according to the manufacturer’s instruction and cultured for 72 h thereafter. Silencer select predesigned siRNAs were purchased from Life Technologies (hsSting siRNA 1, S50644; hsSting siRNA 2, S50646).

### ELISA

Supernatants of human explant tissue and fibroblast cell cultures were collected and centrifuged at 1,000*g* at 4 °C to remove cell debris and dead cells. ELISA was performed according to the instructions of the Human IL-6, IL-8 and MCP-1 ELISA sets from BD Biosciences (BD OpetEIA, 555220, 555244, 555179, respectively).

### 2′3′-cGAMP ELISA

Mouse tissues and cells were lysed in Pierce RIPA Buffer (for tissues, disruption was obtained with the homogenizer (Thermo Fisher Scientific)), and a protease inhibitor cocktail (Sigma-Aldrich, 11836170001) was added to prevent protein degradation. The protein concentration of the lysate was measured using the Pierce BCA Protein Assay (Thermo Fisher Scientific, 23227) to normalize the 2′3′-cGAMP concentrations measured by enzyme-linked immunosorbent assay (ELISA) according to the manufacturer’s instructions (Cayman Chemical, CAY-501700-96).

### Tissue preparation for block face scanning electron microscopy

Adult mice (aged) were perfused, through the heart, with 50 ml of a buffered mix of 1% glutaraldehyde and 2% PFA in 0.1 M phosphate buffer (pH 7.4). The animal was left for 2 h after the perfusion had finished, and the brain was then carefully dissected from the skull and placed into PBS. Next, 80-µm-thick sections were cut with a vibratome, in the sagittal plane, and sections containing the CA1 region of the hippocampus were collected. The sections were then post-fixed in potassium ferrocyanide (1.5%) and osmium (2%), then stained with thiocarbohydrazide (1%) followed by osmium tetroxide (2%). Staining was performed overnight in uranyl acetate (1%) followed by washing in distilled water at 50 °C, and another staining with lead aspartate at the same temperature. The sections were dehydrated in increasing concentrations of ethanol and then embedded in Spurr’s resin and hardened at 65 °C for 24 h between glass slides.

### Block face scanning electron microscopy and analysis

To collect serial electron microscopy images of microglia in the CA1 region of the hippocampus, small (approximately 0.5 mm square) blocks were trimmed from the rest of the section using a razor blade and glued to an aluminium stub using conductive glue. Trimming with a glass knife produced a small (approximately 300 × 300 µm) block that was then mounted inside a scanning electron microscope (Zeiss Merlin, Zeiss NTS) holding a block face cutting microtome (3View, Gatan). Layers of resin, 50 nm thick, were cut from the block surface, and sequential images, targeting microglia, were collected after each layer was removed. An acceleration voltage of 1.7 kV was used with a pixel size of 7 nm and a dwell time of 1 µs. A series of nearly aligned images was collected, and the final alignment was performed using ImageJ. Counts of mitochondria were made through the image series, and each one was scored according to its morphology. The number of those appearing with heavily disrupted membranes and distorted morphologies was counted against those that appeared normal.

### Statistics and reproducibility

Data are presented as mean ± s.e.m. unless otherwise indicated. The sample number (*n*) indicates the number of patients, mice, ROIs or cellular experiment repeats, as specified in the figure legends. We used two-sided Student’s *t*-tests to compare paired or independent samples, one-way analysis of variance (ANOVA) followed by Tukey’s or two-sided ANOVA followed by Sidak’s multiple-comparison tests as indicated and an adjusted *P* < 0.05 was set as the cut-off. Data distribution was assumed to be normal, but this was not formally tested. The experimenters were not blinded to the experimental conditions, and no randomization was performed. Details of the statistical analysis are provided in the corresponding figure legends. Excel v.16.72 was used to combine data from multiple experiments or datasets. Prism v.9.0 was used to generate graphs and calculate statistics using appropriate statistical tests depending on the data, including two-sided paired and unpaired *t*-tests, and one-way or two-way ANOVA. Adjusted *P* values were assessed using appropriate correction methods, such as Tukey, Sidak and Geisser–Greenhouse.

### Reporting summary

Further information on research design is available in the Nature Portfolio Reporting Summary linked to this article.

## Online content

Any methods, additional references, Nature Portfolio reporting summaries, source data, extended data, supplementary information, acknowledgements, peer review information; details of author contributions and competing interests; and statements of data and code availability are available at 10.1038/s41586-023-06373-1.

## Supplementary information

Supplementary Information Supplementary Figs. 1 and 2, containing the uncropped blots and gating strategy used for microglia sorting, and a guide to Supplementary Tables 1–6.

Reporting Summary

Supplementary Table 1 A list of qPCR primers and sgRNA sequences used in the study.

Supplementary Table 2 A list of antibodies used in the study.

Supplementary Table 3 DEGs in the annotated Seurat cell clusters from single-nucleus microglia. Statistical analysis: differential expression was calculated using the MAST statistical framework.

Supplementary Table 4 DEGs between *Tmem119-creER*^*T2*^*-Cgas*^*WT/WT*^ and *Tmem119-creER*^*T2*^*-Cgas*^*WT/R241E*^ mouse microglia in the annotated cell states. Statistical analysis: differential expression was calculated using the MAST statistical framework.

Supplementary Table 5 DEGs between *Tmem119-creER*^*T2*^*-Cgas*^*WT/WT*^and *Tmem119-creER*^*T2*^*-Cgas*^WT/R241E^ mouse microglia in the annotated cell types. Statistical analysis: differential expression was calculated using the MAST statistical framework.

Supplementary Table 6 Gene lists used to define annotated cell states and cell types. Gene sets from existing publications and the Human Protein Atlas single-cell data were used.

## Data Availability

Full scans for all immunoblots are provided in Supplementary Fig. 1. RNA-seq datasets are available at the GEO (GSE234422). Source data are provided with this paper.

## Source data

Source Data Fig. 1
Source Data Fig. 2
Source Data Fig. 3
Source Data Fig. 4
Source Data Extended Data Fig. 1
Source Data Extended Data Fig. 2
Source Data Extended Data Fig. 3
Source Data Extended Data Fig. 4
Source Data Extended Data Fig. 5
Source Data Extended Data Fig. 6
Source Data Extended Data Fig. 7
Source Data Extended Data Fig. 8
Source Data Extended Data Fig. 10
