# Supplementary material for: cGAS–STING drives ageing-related inflammation and neurodegeneration
Source: Nature. 2023 Aug 2;620(7973):374–80. doi: 10.1038/s41586-023-06373-1 (PMC10412454; doi:10.1038/s41586-023-06373-1)

nature portfolio

https://doi.org/10.1038/s41586-023-06373-1

Supplementary information

cGAS–STING drives ageing-related inflammation and neurodegeneration

In the format provided by the authors and unedited

cGAS/STING drives ageing-related inflammation and neurodegeneration

Muhammet F. Gulen[1,9], Natasha Samson[1,9], Alexander Keller[1], Marius Schwabenland[2], Chong Liu[1], Selene Glück[1], Vivek V. Thacker[1], Lucie Favre[3], Bastien Mangeat[4], Lona Kroese[5], Paul Krimpenfort[5], Marco Prinz[2,6,7] & Andrea Ablasser[1,8,*]

[1] Global Health Institute, Swiss Federal Institute of Technology Lausanne (EPFL), Lausanne, Switzerland

[2] Institute of Neuropathology, Faculty of Medicine, University of Freiburg, Freiburg, Germany

[3] Division of Endocrinology, Diabetology, and Metabolism, Lausanne University Hospital, Lausanne, Switzerland

[4] Gene Expression Core Facility, Swiss Federal Institute of Technology Lausanne (EPFL), Lausanne, Switzerland

[5] Animal Modeling Facility, The Netherlands Cancer Institute, The Netherlands

[6] Center for Basics in NeuroModulation (NeuroModulBasics), Faculty of Medicine, University of Freiburg, Freiburg, Germany

[7] Signalling Research Centres BIOSS and CIBSS, University of Freiburg, Freiburg, Germany

[8] Institute for Cancer Research (ISREC), Swiss Federal Institute of Technology Lausanne (EPFL), Lausanne, Switzerland

Supplementary Information Guide

Supplementary Fig. 1. Source data for western blot analysis.
Supplementary Fig. 2. Gating strategy used for microglia sorting.

Supplementary Table 1. qPCR primers and sgRNA sequences used in the study
Tab 1 List of primers and sgRNA sequences (mouse and human-specific).

Supplementary Table 2. Antibodies used in the study
Tab 1 List of antibodies used for immunoblotting, IF, IHC staining, FACS, and neutralization.

Supplementary Table 3. Differentially expressed genes in the annotated Seurat cell clusters from single nuclei microglia
Tab 1 IFN – Differentially expressed genes identifying interferon-associated microglia
Tab 2 DAM-1 – Differentially expressed genes identifying disease-associated microglia, cluster 1
Tab 3 DAM-2 – Differentially expressed genes identifying disease-associated microglia, cluster 2
Tab 4 DAM1&2 – Differentially expressed genes identifying the combined disease-associated microglia of cluster 1 and 2
Tab 5 ND – Differentially expressed genes identifying neurodegenerative-associated microglia
Tab 6 MG1 – Differentially expressed genes identifying homeostatic microglia, cluster 1
Tab 7 MG2 – Differentially expressed genes identifying homeostatic microglia, cluster 2
Tab 8 MG3 – Differentially expressed genes identifying homeostatic microglia, cluster 3

Supplementary Table 4. Differentially expressed genes between *Tmem119-creER*^{T2}*-cGAS*$^{wt/wt}$ and *Tmem119-creER*^{T2}*-cGAS*$^{wt/R241E}$ mice microglia in the annotated cell states

Tab 1 IFN – Differentially expressed genes between *Tmem119-creER*^{T2}*-cGAS*$^{wt/wt}$ and *Tmem119-creER*^{T2}*-cGAS*$^{wt/R241E}$ mice in interferon-associated microglia

Tab 2 DAM-1 – Differentially expressed genes between *Tmem119-creER*^{T2}*-cGAS*$^{wt/wt}$ and *Tmem119-creER*^{T2}*-cGAS*$^{wt/R241E}$ mice in disease-associated microglia, cluster 1

Tab 3 DAM-2 – Differentially expressed genes between *Tmem119-creER*^{T2}*-cGAS*$^{wt/wt}$ and *Tmem119-creER*^{T2}*-cGAS*$^{wt/R241E}$ mice in disease-associated microglia, cluster 2

Tab 4 DAM1&2 – Differentially expressed genes between *Tmem119-creER*^{T2}*-cGAS*$^{wt/wt}$ and *Tmem119-creER*^{T2}*-cGAS*$^{wt/R241E}$ mice in the combined disease-associated microglia, clusters 1 and 2

Tab 5 ND – Differentially expressed genes between *Tmem119-creER*^{T2}*-cGAS*$^{wt/wt}$ and *Tmem119-creER*^{T2}*-cGAS*$^{wt/R241E}$ mice in neurodegenerative-associated microglia

Tab 6 H-MG – Differentially expressed genes between *Tmem119-creER*^{T2}*-cGAS*$^{wt/wt}$ and *Tmem119-creER*^{T2}*-cGAS*$^{wt/R241E}$ mice in homeostatic microglia

Tab 7 All MG – Differentially expressed genes between *Tmem119-creER*^{T2}*-cGAS*$^{wt/wt}$ and *Tmem119-creER*^{T2}*-cGAS*$^{wt/R241E}$ mice across all microglia

Supplementary Table 5. Differentially expressed genes between *Tmem119-creER*^{T2}*-cGAS*$^{wt/wt}$ and *Tmem119-creER*^{T2}*-cGAS*$^{wt/R241E}$ mice microglia in the annotated cell types

Tab 1 Oligodendrocytes – Differentially expressed genes between *Tmem119-creER*^{T2}*-cGAS*$^{wt/wt}$ and *Tmem119-creER*^{T2}*-cGAS*$^{wt/R241E}$ mice in oligodendrocytes

Tab 2 Astrocytes – Differentially expressed genes between *Tmem119-creER*^{T2}*-cGAS*$^{wt/wt}$ and *Tmem119-creER*^{T2}*-cGAS*$^{wt/R241E}$ mice in astrocytes

Tab 3 Microglia – Differentially expressed genes between *Tmem119-creER*^{T2}*-cGAS*$^{wt/wt}$ and *Tmem119-creER*^{T2}*-cGAS*$^{wt/R241E}$ mice in microglia

Tab 4 Neurons – Differentially expressed genes between *Tmem119-creER*^{T2}*-cGAS*$^{wt/wt}$ and *Tmem119-creER*^{T2}*-cGAS*$^{wt/R241E}$ mice in neurons

Supplementary Table 6. Gene lists used to define annotated cell states and cell types

Tab 1 H MG – Homeostatic microglia markers

Tab 2 ND MG genes – Neurodegenerative-associated microglia markers

Tab 3 DAM genes – Disease-associated microglia markers

Tab 4 IFN genes – Interferon-associated microglia markers

Tab 5 Oligodendrocytes – Oligodendrocytes specific markers

Tab 6 Astrocytes – Astrocyte specific markers

Tab 7 Neurons – Neuron specific markers

Tab 8 Microglia – Microglia specific markers

Supplementary Table Legends

Supplementary Fig. 1 I Source data for western blot analysis. Uncropped Western blot raw data. The molecular weight marker is indicated on the left (given in kDa). Boxes in dotted lines indicate cropped regions as presented in the figures and the extended data figures.

Supplementary Fig. 2 I Gating strategy used for microglia sorting. To enrich microglial cells, in addition to DAPI staining, nuclei were stained with anti-RBFOX3/NeuN-647 and anti-Olig2-488, and DAPI^{+}/NeuN^{-}/Olig2^{-} cells were sorted for analysis.

Supplementary Table 3 I Differentially expressed genes in the annotated Seurat cell clusters from single nuclei microglia. Statistical analysis: Differential expression was calculated using the MAST statistical framework.

Supplementary Table 4 I Differentially expressed genes between *Tmem119-creER^{T2}-cGAS$^{wt/wt}$* and *Tmem119-creER^{T2}-cGAS$^{wt/R241E}$* mice microglia in the annotated cell states. Statistical analysis: Differential expression was calculated using the MAST statistical framework.

Supplementary Table 5 I Differentially expressed genes between *Tmem119-creER^{T2}-cGAS$^{wt/wt}$* and *Tmem119-creER^{T2}-cGAS$^{wt/R241E}$* mice microglia in the annotated cell types. Statistical analysis: Differential expression was calculated using the MAST statistical framework.

Supplementary Table 6 I Gene lists used to define annotated cell states and cell types. Gene sets from existing publications and the Human Protein Atlas single-cell data were used.

Supplementary Figure 1: Source data for western blot analysis. Uncropped Western blot raw data. The molecular weight marker is indicated on the left (given in kDa). Boxes in dotted lines indicate cropped regions as presented in the figures and the extended data figures.

Fig 2d

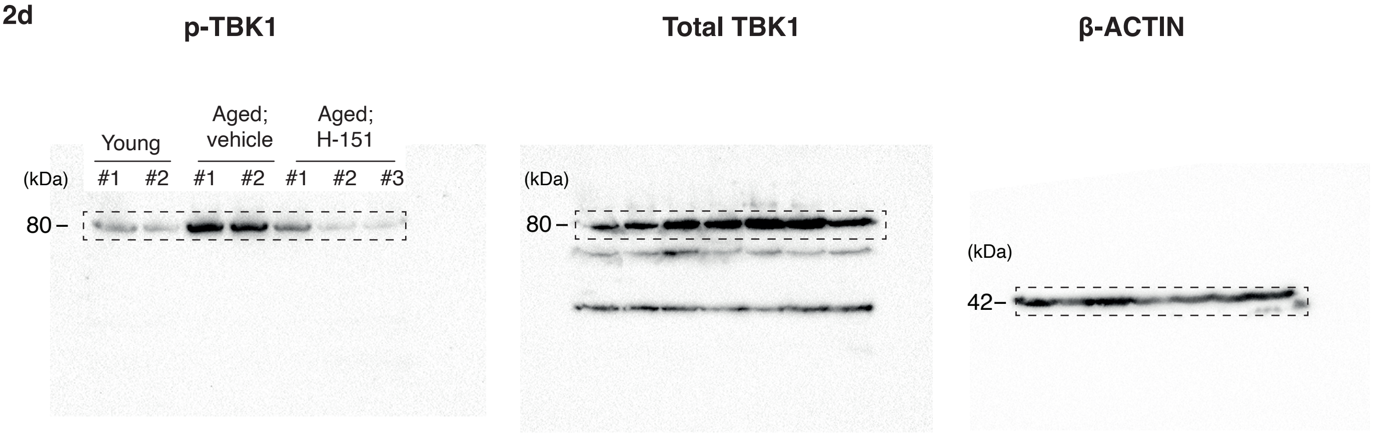

Fig Ext. 2d

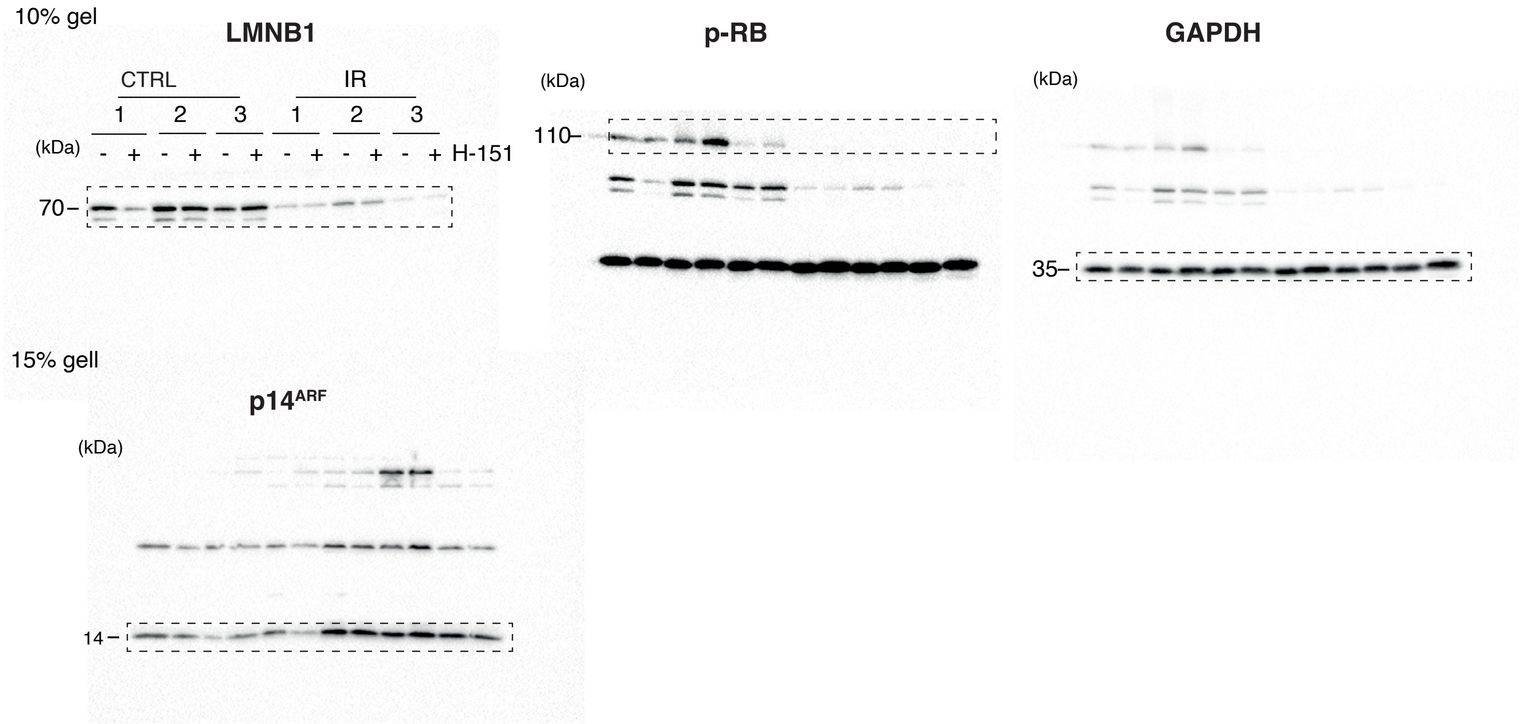

Fig Ext. 7b

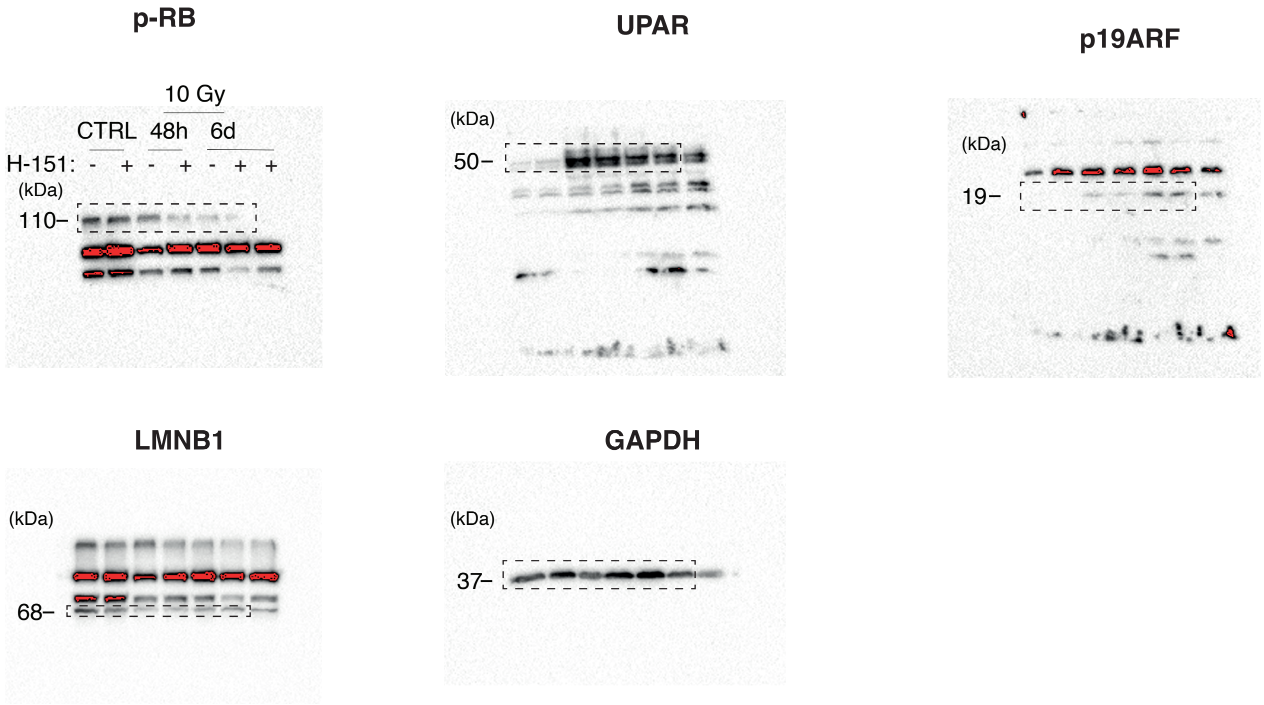

Fig Ext. 8b

p-TBK1

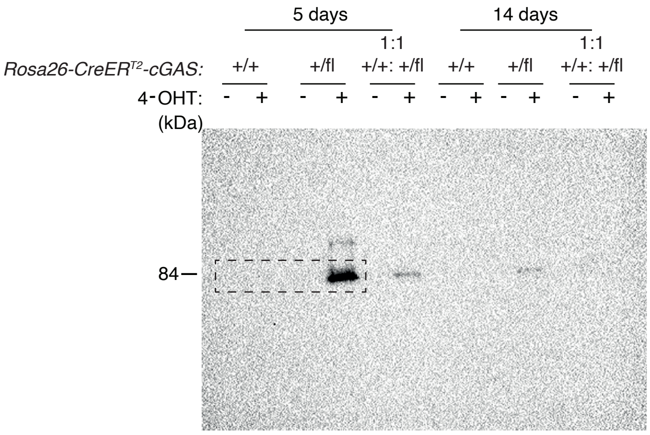

Total TBK1

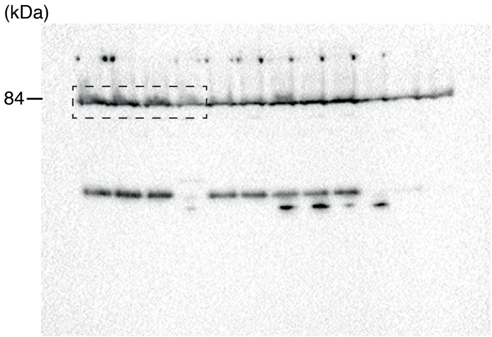

Total STING

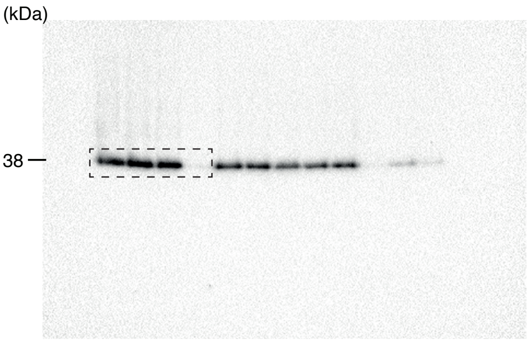

p-STING

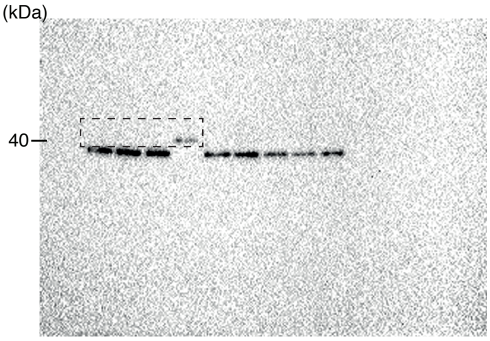

Vinculin

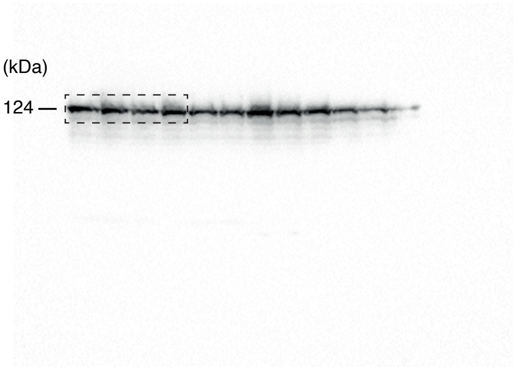

Supplementary Figure 2: Gating strategy used for microglia sorting. To enrich microglial cells, in addition to DAPI staining, nuclei were stained with anti-RBFOX3/NeuN-647 and anti-Olig2-488, and DAPI+/NeuN-/Olig2- cells were sorted for analysis.

Gating Strategy for Single Nucleus RNA sequencing experiments

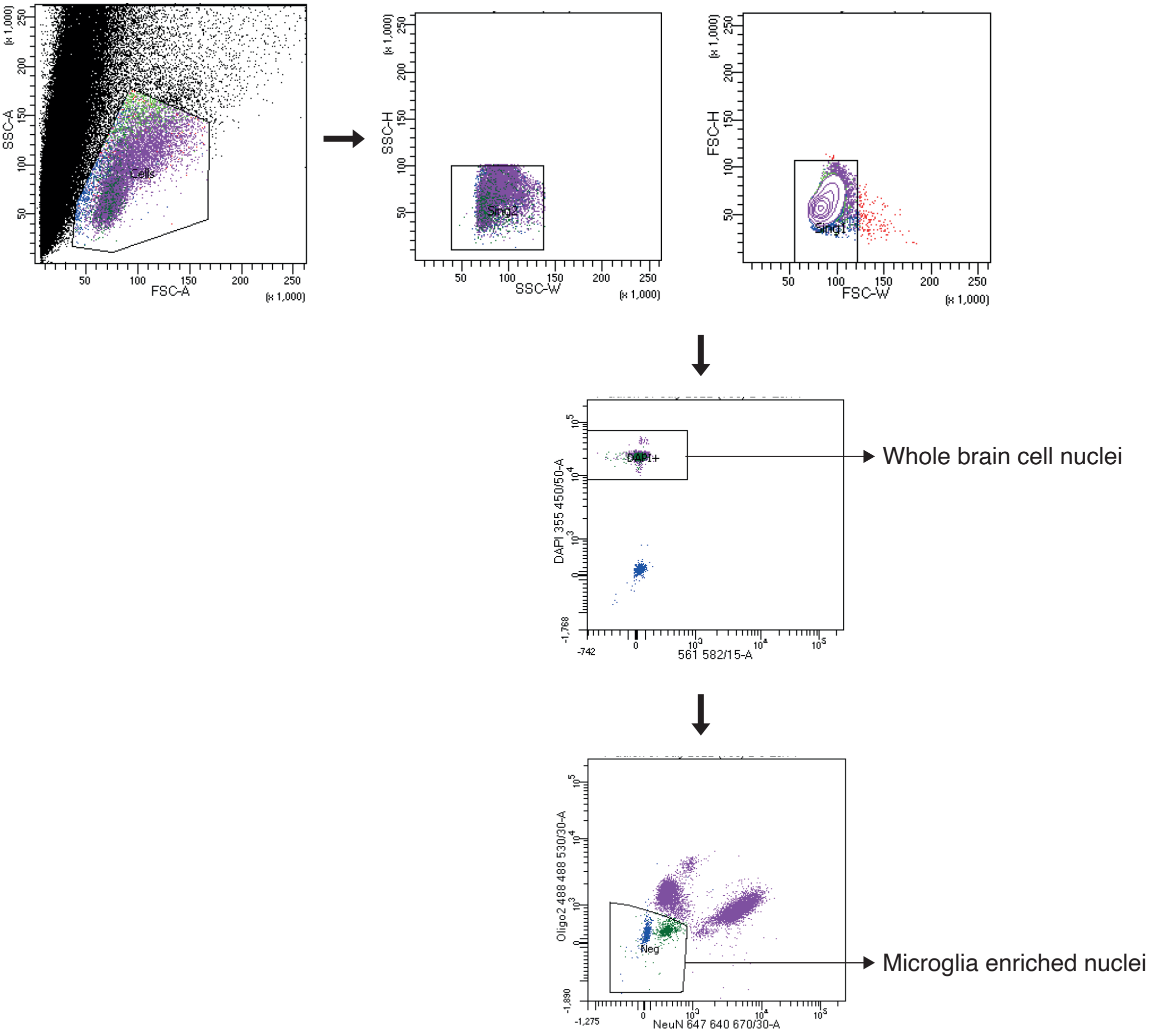

Supplement: Supplementary file 1 — Supplementary Figs. 1 and 2, containing the uncropped blots and gating strategy used for microglia sorting, and a guide to Supplementary Tables 1–6. [file 41586_2023_6373_MOESM1_ESM.pdf]
