# Supplementary material for: cGAS–STING drives ageing-related inflammation and neurodegeneration
Source: Nature. 2023 Aug 2;620(7973):374–80. doi: 10.1038/s41586-023-06373-1 (PMC10412454; doi:10.1038/s41586-023-06373-1)
Supplement: Supplementary file 2 — Reporting Summary [file 41586_2023_6373_MOESM2_ESM.pdf]

nature research

Corresponding author(s): Andrea Ablasser

Last updated by author(s): Jun 8, 2023

Reporting Summary

Nature Research wishes to improve the reproducibility of the work that we publish. This form provides structure for consistency and transparency in reporting. For further information on Nature Research policies, see our Editorial Policies and the Editorial Policy Checklist.

Statistics

For all statistical analyses, confirm that the following items are present in the figure legend, table legend, main text, or Methods section.

n/a	Confirmed	
☐	☒	The exact sample size (n) for each experimental group/condition, given as a discrete number and unit of measurement
☐	☒	A statement on whether measurements were taken from distinct samples or whether the same sample was measured repeatedly
☐	☒	The statistical test(s) used AND whether they are one- or two-sided<br>*Only common tests should be described solely by name; describe more complex techniques in the Methods section.*
☒	☐	A description of all covariates tested
☐	☒	A description of any assumptions or corrections, such as tests of normality and adjustment for multiple comparisons
☐	☒	A full description of the statistical parameters including central tendency (e.g. means) or other basic estimates (e.g. regression coefficient) AND variation (e.g. standard deviation) or associated estimates of uncertainty (e.g. confidence intervals)
☐	☒	For null hypothesis testing, the test statistic (e.g. F, t, r) with confidence intervals, effect sizes, degrees of freedom and P value noted<br>*Give P values as exact values whenever suitable.*
☒	☐	For Bayesian analysis, information on the choice of priors and Markov chain Monte Carlo settings
☒	☐	For hierarchical and complex designs, identification of the appropriate level for tests and full reporting of outcomes
☒	☐	Estimates of effect sizes (e.g. Cohen's d, Pearson's r), indicating how they were calculated

Our web collection on statistics for biologists contains articles on many of the points above.

Software and code

Policy information about availability of computer code

Data collection	Western blots were acquired using Image Lab software (Biorad). RT-qPCR data were acquired using QuantStudio (5, 6 or 7) Real-Time PCR system (ThermoFisher). Microscopic images were aquired using Axiovision software using Axioplan (Zeiss), Olympus OlyVIA software using Olympus V120 whole slide scanner, Zeiss LSM 700 Inverted microscope, Leica SP8 confocal microscope. Super-resolution Airyscan images were acquired on a Zeiss LSM 980 with Airyscan microscope (Carl Zeiss) and data was collected Zeiss ZEN software. Western Blot images were analysed using Image Lab (Biorad). ECL signal was recorded using ChemiDoc XRS Biorad Imager.
Data analysis	GraphPad PRISM 9, Microsoft Excel 16.72<br>Single cell sequencing was processed using 10X Genomics Cell Ranger Single Cell Software Suite v7.0.0.<br>R (R v4.0 & , 4.2.2), R-Studio (2022.07.1 Build 554), R packages: Seurat (4.1.1.9003), MAST (1.22.0), scCustomize (0.7.0), ComplexHeatmap (2.12.1), EnhancedVolcano (1.13.2)<br>Images were analysed and rendered using FIJI with Image J 1.53. Leica SP8 confocal microglia z-stacks were subsequently deconvolved using the Huygens Deconvolution Software (Scientific Volume Imaging), and 3D views were rendered using Bitplane Imaris 9.6.

For manuscripts utilizing custom algorithms or software that are central to the research but not yet described in published literature, software must be made available to editors and reviewers. We strongly encourage code deposition in a community repository (e.g. GitHub). See the Nature Research guidelines for submitting code & software for further information.

Data

Policy information about availability of data

All manuscripts must include a data availability statement. This statement should provide the following information, where applicable:

- Accession codes, unique identifiers, or web links for publicly available datasets
- A list of figures that have associated raw data
- A description of any restrictions on data availability

The datasets generated during and/or analysed during the current study are available from the corresponding authors on reasonable request.
Full scans for all western blots images are provided in Supplementary Fig. 1. Source data for all data are shown in the corresponding "Source Data" file. All other data are available from the corresponding author on reasonable request.

RNA-seq datasets are made available through the Gene Expression Omnibus (GEO) depository (GSE234422). Datasets used for comparison to aged and diseased microglia were acquired from GEO (GSM4505405 and GSE127892, respectively). Builds of the human and mouse genomes used in this study (hg38, GRCm39, mm10) are publicly available through ensembl or NCBI.

The datasets generated during and/or analysed during the current study & all other dat..

Field-specific reporting

Please select the one below that is the best fit for your research. If you are not sure, read the appropriate sections before making your selection.

☒ Life sciences ☐ Behavioural & social sciences ☐ Ecological, evolutionary & environmental sciences

For a reference copy of the document with all sections, see nature.com/documents/nr-reporting-summary-flat.pdf

Life sciences study design

All studies must disclose on these points even when the disclosure is negative.

Sample size	We have used at least three biological replicates for each experiment unless stated otherwise. This is consistent with previous studies and accounts for biological variability between distinct samples from inbred mice or cell lines. Although we did not use statistical methods to calculate sample size, we used a minimum of 3 biological replicates. For mice experiments, we based our numbers on previously published experiments where differences were observed, taking into account the 3 Rs principle and the fact that the majority of experiments were performed in inbred mice. See the statistical analysis sections of methods for full details.
Data exclusions	Extended Data Fig. 3<br>The data used is representative of 11 out of total 18 patients' white adipose tissue explants analyzed. The 7 patients that were not included were either showing highly increased inflammatory markers compared to the majority of patients analyzed and therefore considered as outliers, or only showing a minor increase in inflammation as compared to the senolytic control D+Q, in which case H-151 treatment was not showing a suppressive effect.<br>Fig. 4, Extended Fig. 9<br>One cGas-wt/R241E mouse was excluded from analysis as the genotype of the mouse was incorrect.
Replication	Experimental findings were reliably reproduced. The number (n) of biological replicates or animals is indicated as an exact number in the figure legends.
Randomization	For in vivo studies, mice were randomly assigned to the treatment groups. All in vitro treatment groups were randomly assigned.
Blinding	In vitro and in vivo experiments were not blinded due to lack of available experimenters with required expertise or because the experimental conditions were evident from image data. The experimenters were blinded for the analysis of the histopathological scores for experimental groups.

Reporting for specific materials, systems and methods

We require information from authors about some types of materials, experimental systems and methods used in many studies. Here, indicate whether each material, system or method listed is relevant to your study. If you are not sure if a list item applies to your research, read the appropriate section before selecting a response.

Materials & experimental systems

n/a	Involved in the study	
☐	☒	Antibodies
☐	☒	Eukaryotic cell lines
☒	☐	Palaeontology and archaeology
☐	☒	Animals and other organisms
☐	☒	Human research participants
☒	☐	Clinical data
☒	☐	Dual use research of concern

Methods

n/a	Involved in the study	
☒	☐	ChIP-seq
☒	☐	Flow cytometry
☒	☐	MRI-based neuroimaging

Antibodies

Antibodies used

Primary antibodies:
Rabbit anti-GAPDH (14C10) Cell Signaling #2118
Mouse anti-beta-Actin Santa Cruz s-47778
Rabbit anti-Vinculin Sigma V9264
Rabbit anti-phospho-RB (Ser807/611) D20B12 Cell Signaling #8516
Rabbit anti-LaminB1 D4Q4Z Cell Signaling 125865
Rabbit anti-phospho-TBK/NAK (Ser172) D52C2 Cell Signaling #5483
Rabbit anti-TBK1 Novus Bio NB100-56705
Rabbit anti-human-phospho-STING(Ser366) D8K6H BioConcept #40818
Rabbit anti-mouse-phospho-STING(Ser365) D1C4T BioConcept #62912
Rabbit anti-STING D2P2F Cell Signaling #13547
Rabbit anti-p21/CIP1/CDKN1A(pThr145) Novus Bio NB100-92599
Rabbit anti-human-p21 Cell Signaling #2947
Goat anti-uPAR Bio-Techne AF534-SP
Rat anti-F4/80 A3-1 Thermo Fisher Scientific MA1-91124
Mouse anti-MAP2 Sigma-Aldrich M4403, IF used 1:250
Rabbit anti-IBA1 Abcam ab178846, used 1:10000
Goat anti-IBA1 Abcam ab5076, IF used 1:300
Rabbit anti-Mac3 Becton Dickinson 553322, used 1:150
Rabbit anti-GFAP Aglient Z033429-2(AGI), used 1:100
Mouse anti-NeuN Merck MAB377, used 1:100
Rabbit anti-synaptophysin Cell Signaling 36406S, used 1:500
Rabbit anti-B2M Abcam ab75853, IF used 1:100
Mouse anti-dsDNA Merc MAB1293, IF used 1:500
Rabbit anti-TOMM20 Abcam ab232589, IF used 1:500

Neutralizing Antibodies:
Mouse anti-IFNAR1 Sigma-Aldrich MARI-5A3, used 1:1000
Rat anti-TNFa XT3.11 Bio X Cell BE0058, used 1:250

Antibodies for FACS sorting of nuclei
Mouse RBFOX3/NeuN-647 1B7 Novus Biologicals NBP1-92693A, used 1:100
Mouse Olig2-488 211F1.1 Merck MABN50A4, used 1:100

Secondary antibodies:
Donkey anti-rabbit IgG (H+L) AF568 Invitrogen A10042
Donkey anti-rabbit IgG (H+L) AF488 Invitrogen A21206
Goat anti-mouse IgG (H+L) AF488 Invitrogen A11029
Donkey anti-Goat IgG (H+L) AF488 Invitrogen A11055
Goat anti-mouse IfF (H+L) AF568 Invitrogen A10037
Donkey Anti-Rabbit-HRP Jackson ImmunoResearch 711035152
Donkey Anti-Mouse-HRP Jackson ImmunoResearch 715035150

Validation

Primary Antibodies have been validated for use for immunofluorescence by the manufacturers as stated on their respective websites. Antibodies were used according to the validation listed in the manufacturer's instructions (details of antibody validation are given in Table S2). Aliquots of secondary antibodies were provided by the Histology Core Facility at EPFL and have been validated by the manufacturers.

Eukaryotic cell lines

Policy information about cell lines

Cell line source(s)

ATCC (WI-38, BJ), AMSBio (BV2)
- WI-38 ATCC CCL-75

	- BJ 5ta ATCC CRL-4001<br>- BV2 AMS.EP-CL-0493
Authentication	The identity of the cell lines was verified by the supplier (ATCC for WI-38 and BJ; AMSBio for BV-2). In addition, cell line authentication was performed based on their characteristic morphology.
Mycoplasma contamination	Cells were repeatedly tested for mycoplasma using specific primers and always found to be negative as compared to the positive control.
Commonly misidentified lines<br>(See ICLAC register)	none

Animals and other organisms

Policy information about studies involving animals; ARRIVE guidelines recommended for reporting animal research

Laboratory animals	In this study, wild-type C57BL/6J mice and Tmem173-/- (STING-deficient) (Strain#: 025805), B6;129Gt(ROSA)26Sortm1(cre/ERT)Nat/J (The Jackson Laboratory, Strain#: 004847) and C57BL/6-Tmem119em1(cre/ERT2)Gfng/J (The Jackson Laboratory, Strain#: 031820) mice were purchased from The Jackson Laboratory. The cGasR241E mice were generated in the Netherlands Cancer Institute, as described in the Methods. At the time of sample collection, young mice were between 8-12 weeks of age, aged mice were around 26 months old, as mentioned in the manuscript.<br>Mice were housed in groups of up to 5 mice/cage at 18 degrees C-24 degrees C ambient temperatures with 40-60% humidity. Mice were maintained on a 12 hour light/ dark cycle 6 am to 6 pm. Food and water were available ad libitum. Aged mice were single caged to avoid aggressive behavior-induced injury.
Wild animals	The study did not involve wild animals
Field-collected samples	The study did not involve samples collected from the field
Ethics oversight	Animal experiments were approved by the Service de la Consommation et des Affaires Vétérinaires of the canton of Vaud (Switzerland) and were performed in accordance with the respective legal regulations.

Note that full information on the approval of the study protocol must also be provided in the manuscript.

Human research participants

Policy information about studies involving human research participants

Population characteristics	Human adipose tissue was resected during gastric bypass surgery from obese subjects. Three were male, and eight were female. The mean age of the subjects was: mean 48.7 y.o.; s.d 9.7; range 30-60. No subject was known to have a malignancy.
Recruitment	All material used in this study was obtained from the Cohort of Obese Patients of Lausanne with an ethically approved license by the commission of Vaud Canton (CER-VD Project PB_2018-00119). The coded samples were collected under signed informed consent conforming to the guidelines of the 2000 Helsinki declaration. No selection bias was noted.
Ethics oversight	Studies were approved by the University Hospital of Lausanne (CHUV) and were performed in accordance with the guidelines of the Declaration of Helsinki and were reviewed by the ethical committee board of the canton of Vaud (CER-VD 2020-02204).

Note that full information on the approval of the study protocol must also be provided in the manuscript.
